# Unbiased Metagenomic Sequencing for Pediatric Meningitis in Bangladesh Reveals Neuroinvasive Chikungunya Virus Outbreak and Other Unrealized Pathogens

**DOI:** 10.1128/mBio.02877-19

**Published:** 2019-12-17

**Authors:** Senjuti Saha, Akshaya Ramesh, Katrina Kalantar, Roly Malaker, Md Hasanuzzaman, Lillian M. Khan, Madeline Y. Mayday, M. S. I. Sajib, Lucy M. Li, Charles Langelier, Hafizur Rahman, Emily D. Crawford, Cristina M. Tato, Maksuda Islam, Yun-Fang Juan, Charles de Bourcy, Boris Dimitrov, James Wang, Jennifer Tang, Jonathan Sheu, Rebecca Egger, Tiago Rodrigues De Carvalho, Michael R. Wilson, Samir K. Saha, Joseph L. DeRisi

**Affiliations:** a Child Health Research Foundation, Department of Microbiology, Dhaka Shishu Hospital, Dhaka, Bangladesh; b Department of International Health, Johns Hopkins Bloomberg School of Public Health, Baltimore, Maryland, USA; c Weill Institute for Neurosciences, University of California, San Francisco, California, USA; d Department of Neurology, University of California, San Francisco, California, USA; e Department of Biochemistry and Biophysics, University of California, San Francisco, California, USA; f UCSF School of Medicine, Benioff Children’s Hospital, Department of Pediatrics, Division of Critical Care, San Francisco, California, USA; g Chan Zuckerberg Biohub, San Francisco, California, USA; h Division of Infectious Diseases, Department of Medicine, University of California, San Francisco, California, USA; i Department of Microbiology and Immunology, University of California, San Francisco, California, USA; j Chan Zuckerberg Initiative, Redwood City, California, USA; k Bangladesh Institute of Child Health, Dhaka Shishu Hospital, Dhaka, Bangladesh; Virginia Polytechnic Institute and State University

**Keywords:** idiopathic meningitis, metagenomics, etiology, Bangladesh, cerebrospinal fluid, Chikungunya virus, meningitis, virology

## Abstract

The burden of meningitis in low-and-middle-income countries remains significant, but the infectious causes remain largely unknown, impeding institution of evidence-based treatment and prevention decisions. We conducted a validation and application study of unbiased metagenomic next-generation sequencing (mNGS) to elucidate etiologies of meningitis in Bangladesh. This RNA mNGS study was performed on cerebrospinal fluid (CSF) specimens from patients admitted in the largest pediatric hospital, a World Health Organization sentinel site, with known neurologic infections (*n* = 36), with idiopathic meningitis (*n* = 25), and with no infection (*n* = 30), and six environmental samples, collected between 2012 and 2018. We used the IDseq bioinformatics pipeline and machine learning to identify potentially pathogenic microbes, which we then confirmed orthogonally and followed up through phone/home visits. In samples with known etiology and without infections, there was 83% concordance between mNGS and conventional testing. In idiopathic cases, mNGS identified a potential bacterial or viral etiology in 40%. There were three instances of neuroinvasive Chikungunya virus (CHIKV), whose genomes were >99% identical to each other and to a Bangladeshi strain only previously recognized to cause febrile illness in 2017. CHIKV-specific qPCR of all remaining stored CSF samples from children who presented with idiopathic meningitis in 2017 (*n* = 472) revealed 17 additional CHIKV meningitis cases, exposing an unrecognized meningitis outbreak. Orthogonal molecular confirmation, case-based clinical data, and patient follow-up substantiated the findings. Case-control CSF mNGS surveys can complement conventional diagnostic methods to identify etiologies of meningitis, conduct surveillance, and predict outbreaks. The improved patient- and population-level data can inform evidence-based policy decisions.

## Abstract (precis)

Globally, there are an estimated 10.6 million cases of meningitis and 288,000 deaths every year, with the vast majority occurring in low- and middle-income countries. In addition, many survivors suffer from long-term neurological sequelae. Most laboratories assay only for common bacterial etiologies using culture and directed PCR, and the majority of meningitis cases lack microbiological diagnoses, impeding institution of evidence-based treatment and prevention strategies. We report here the results of a validation and application study of using unbiased metagenomic sequencing to determine etiologies of idiopathic (of unknown cause) cases. This included CSF from patients with known neurologic infections, with idiopathic meningitis, and without infection admitted in the largest children’s hospital of Bangladesh and environmental samples. Using mNGS and machine learning, we identified and confirmed an etiology (viral or bacterial) in 40% of idiopathic cases. We detected three instances of Chikungunya virus (CHIKV) that were >99% identical to each other and to a strain previously recognized to cause systemic illness only in 2017. CHIKV qPCR of all remaining stored 472 CSF samples from children who presented with idiopathic meningitis in 2017 at the same hospital uncovered an unrecognized CHIKV meningitis outbreak. CSF mNGS can complement conventional diagnostic methods to identify etiologies of meningitis, and the improved patient- and population-level data can inform better policy decisions.

## INTRODUCTION

Globally, there are an estimated 10.6 million cases of meningitis and 288,000 deaths every year, with the vast majority occurring in low- and middle-income countries (LMICs) (1–3). In addition, at least a quarter of survivors suffer from long-term neurological sequelae (4). Several studies report that a significant proportion of meningitis cases lack microbiological diagnoses, and the rate of meningitis with unknown etiology can be as high as 85% in some settings (5, 6). More than 100 bacterial and viral pathogens are known to cause meningitis, but most microbiology laboratories in resource-poor settings test primarily for bacterial pathogens, namely, Neisseria meningitidis, Streptococcus pneumoniae, Haemophilus influenzae, and group B S*treptococcus*, using culture, directed PCR, and/or serology (5, 7). In a World Health Organization (WHO)-supported meningitis surveillance study in Dhaka, Bangladesh (8), we collected 23,140 cerebrospinal fluid (CSF) samples from patients with suspected meningitis between 2004 and 2016, 8,125 of which contained ≥10 white blood cells (WBC)/μl. We were able to detect a bacterial etiology in only 1,585 (20%) of these cases despite the use of multiple diagnostic tools, including culture, antigen, and pathogen-specific qPCR assays. Such low rates of etiology detection hamper implementation of evidence-based policy decisions for optimizing local empirical treatment protocols and disease prevention strategies (5, 9).

The challenges of obtaining a microbiological diagnosis may be due to a combination of multiple factors, including the following: (i) meningitis is caused by a wide variety of microbes, some of which are uncommon and lack diagnostic assays; (ii) prior antibiotic exposure and delay in care-seeking can lower the yield of culture and PCR-based methods; and/or (iii) noninfectious causes of inflammation can mimic infectious meningitis. Drawing on recent studies demonstrating the promise of unbiased metagenomic next-generation sequencing (mNGS) approaches to identify pathogens in diverse biological specimens (10–13), we sought to conduct a retrospective case-control study to investigate CSF of children with idiopathic meningitis in Bangladesh.

## RESULTS

**FIG 1:**
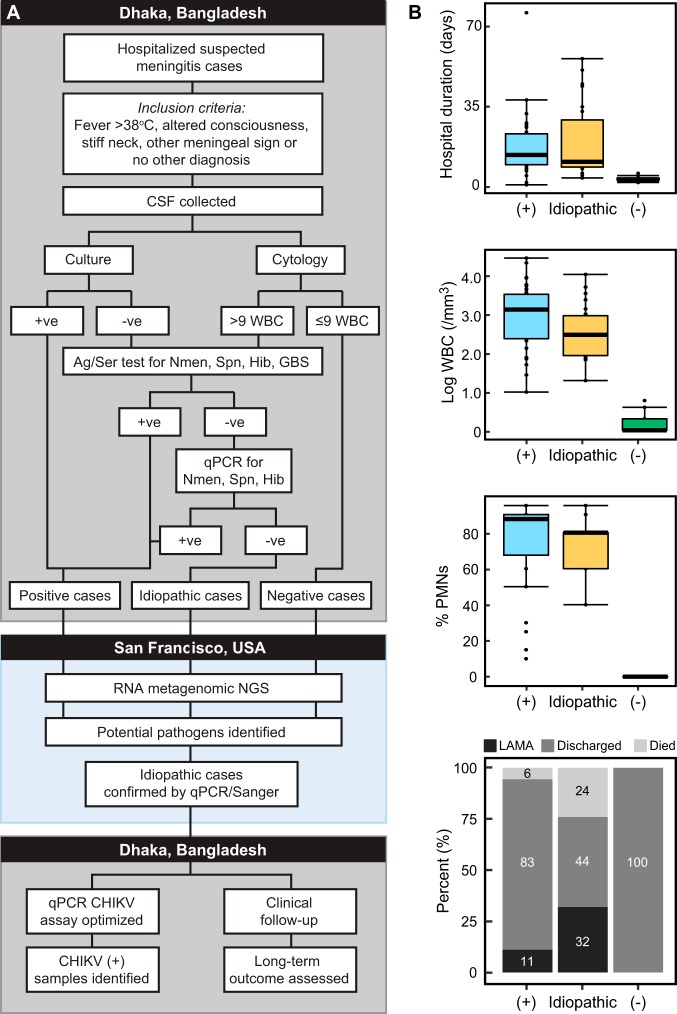
Selection and characteristics of samples used in this study. (A) Study flow diagram. (B) Comparison of clinical characteristics between the three types of samples, positive (+), idiopathic, and negative (−) chosen for this study. Ag, antigen; Ser, serology; Spn, Streptococcus pneumoniae; Nmen, Neisseria meningitidis; Hib, Haemophilus influenzae type b; GBS, group B *Streptococcus*; CHIKV, Chikungunya virus; PMNs, polymorphonuclear neutrophils; +ve, positive; -ve, negative; LAMA, left against medical advice.

CSF specimens were tested from 91 patients (42% female), ranging in age from 0 to 160 months (median: 9 months). Comparative clinical characteristics of the three case types, infectious (*n* = 36), idiopathic (*n* = 25), and noninfectious (*n* = 30), are provided in Fig. 1B and Table S3 in the supplemental material. The majority (71%, *n* = 65) of patients originated in Dhaka division, while the remaining cases sought care from 11 outlying districts (Fig. S2A and B).

**FIG 2:**
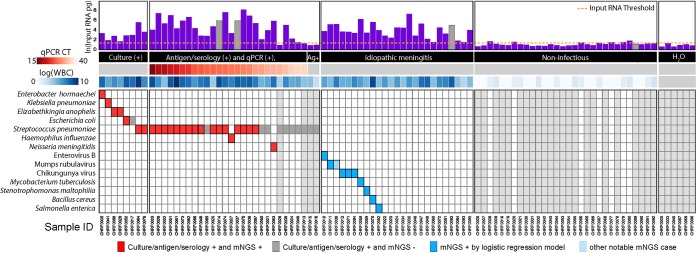
Pathogen identification through mNGS and logistic regression in all sample types. Total input RNA (log pg) is shown for all samples. Samples for which the input RNA values could not be reliably calculated (outliers) are shown as gray bars with imputed input RNA values based on the mean value in their respective groups (known infection, no infection, idiopathic samples). Samples in the known infection group are ordered by increasing cycle threshold, depicted as a heatmap below the *x* axis. Next, the WBC counts obtained by the clinical lab are also plotted as a heatmap. The predicted pathogens for all samples are shown as filled-in squares. Gray squares indicate samples which were considered positive by clinical diagnostic but for which no pathogen was detected by the pathogen-calling algorithm using mNGS data. Red boxes indicate concordant findings, and blue boxes indicate new putative pathogens identified by mNGS data that were not identified by standard clinical methods. The light blue squares indicate pathogens that were not picked up by the logistic regression method but were flagged as potentially interesting by manual review and followed up as if detected. Ag+, antigen positive.

mNGS libraries (n = 97) were prepared and sequenced, resulting in an average depth of 72 million reads/sample (interquartile range [IQR]: 53 to 86 million). The resulting fastq files were processed using the open-source IDseq platform (Fig. S1 and Text S1). Using External RNA Controls 103 Consortium collection (ERCC) spike-in control RNAs, the calculated RNA input masses were highly correlated with WBC count (log-scaled Pearson *r* = 0.69) (14). RNA input masses for the noninfectious CSF samples and template-free controls were significantly less than both the samples of known and unknown etiology (2 pg versus 273 pg, *P* < 0.0001 by Wilcoxon rank sum) (Fig. 2 and Table S5).

10.1128/mBio.02877-19.1 TEXT S1 Bioinformatic analysis and pathogen identification. Download Text S1, PDF file, 0.1 MB. Copyright © 2019 Saha et al. 2019 Saha et al. This content is distributed under the terms of the Creative Commons Attribution 4.0 International license.

10.1128/mBio.02877-19.3 FIG S1 Schematic representation of the bioinformatic approach in IDseq for pathogen identification. Download FIG S1, PDF file, 0.1 MB. Copyright © 2019 Saha et al. 2019 Saha et al. This content is distributed under the terms of the Creative Commons Attribution 4.0 International license.

### Meningitis with known etiology (*n* = 36).

Positive-control samples were drawn from cases with previously identified pathogens, via a combination of standard lab diagnostics, including culture (*n* = 8), qPCR and antigen (*n* = 26), and antigen-only (*n* = 2). Using logistic regression, we correctly identified pathogens in 7 of 8 (88%) samples that were culture positive (Fig. 2). For specimens that were previously PCR and antigen or serology positive, mNGS identified 24 of 27 (89%) samples whose confirmatory qPCR had a cycle threshold (*C_T_*) of <32. Taking into account all specimens that were culture, PCR, and/or antigen-positive (*n* = 36) regardless of *C_T_* value, 25 (69%) specimens were classified as containing potential pathogens by mNGS.

### Noninfectious CSF specimens (*n* = 36).

Among the noninfectious specimens, only 4 (11%) had an input RNA mass greater than 3.0 pg. No potential pathogens were identified in these samples.

### Idiopathic meningitis specimens (*n* = 25).

Potential pathogens were identified in 10 of 25 (40%) idiopathic cases: four cases with bacterial pathogens (Salmonella enterica, Stenotrophomonas maltophilia, Bacillus cereus, and Mycobacterium tuberculosis) and six with viral pathogens (Chikungunya virus [CHIKV], *n* = 3; mumps virus, *n* = 2; and enterovirus B, *n* = 1) (Fig. 2 and Table 1). These cases were further confirmed with orthogonal PCR testing, Sanger sequencing, investigation of case histories, and patient follow-up.

**TABLE 1:** Summary of clinical characteristics of idiopathic cases^*a*^ (*n* = 25)

Sample ID	Age (mo)/sex	WBC/μl (% PMN)/ protein (mg/dl)	Pathogen detected	Provisional/final diagnosis	Hospital duration (days)/outcome	Long-term outcome
Resolved						
CHRF0010	7/M	314 (80)/40	Enterovirus B	Meningitis/meningitis	6/Dis	Healthy
CHRF0058	160/F	360 (80)/200	M. tuberculosis	ICSOL/ICSOL	15/LAMA	Sequelae
CHRF0070	0/M	12,000 (95)/500	B. cereus	Sepsis	15/Dis	Healthy
CHRF0082	4/M	90 (70)/600	S. enterica	Meningitis/meningitis	23/Dis	Healthy
CHRF0094	0/F	1,000 (90)/220	CHIKV	Meningitis/meningitis	10/Dis	Healthy
CHRF0011	18/M	100 (60)/60	Mumps virus	Meningitis/meningitis	9/Dis	Healthy
CHRF0035	21/F	600 (90)/700	Human herpesvirus 6	Acute stroke syndrome/ acute stroke syndrome	56/Died	Died
CHRF0059	4/F	120 (80)/300	S. maltophilia	Meningitis/meningitis	28/Dis	NA
CHRF0071	1/F	180 (80)/250	CHIKV	Meningitis/meningitis	6/Dis	Healthy
CHRF0012	86/F	180 (60)/55	CHIKV	Acute glomerulonephritis/ meningoencephalitis	45/Died	Died
CHRF0036	156/M	1,500 (60)/160	Mumps virus	Meningitis/meningitis	10/LAMA	Healthy

Unresolved						
CHRF0081	72/M	1,100 (40)/350	NA	Meningitis/meningitis	37/LAMA	Died
CHRF0093	13/M	20 (80)/80	NA	Heart disease/ dextrocardia	11/Died	Died
CHRF0022	3/M	460 (80)/300	NA	Meningitis/meningitis	9/LAMA	Died
CHRF0034	23/F	70 (70)/150	NA	Meningitis/meningitis	11/Dis	Sequelae
CHRF0046	1/F	160 (80)/70	NA	Pneumonia/pneumonia	4/LAMA	Healthy
CHRF0023	4/M	74 (70)/100	NA	Meningitis/meningitis	8/Dis	Sequelae
CHRF0047	16/M	2,600 (90)/400	NA	ARI/meningoencephalitis	5/Died	Died
CHRF0083	7/M	5,600 (80)/1,000	NA	Hydrocephalus/pneumonia	10/Died	Died
CHRF0095	98/M	90 (70)/60	NA	Meningoencephalitis/ tubercular meningitis	33/LAMA	Died
CHRF0024	96/F	460 (60)/200	NA	Meningitis/meningitis	8/LAMA	NA
CHRF0048	2/M	3,800 (80)/400	NA	Meningitis/meningitis	35/Dis	NA
CHRF0060	4/M	84 (70)/150	NA	Meningitis/meningitis	11/LAMA	Died
CHRF0072	3/F	900 (60)/300	NA	Meningitis/meningitis	44/Dis	Sequelae
CHRF0084	4/F	70 (60)/250	NA	Meningitis/meningitis	51/Died	Died

a Number of cases is 25. Abbreviations: NA, not available; LAMA, left against medical advice; Dis, discharged; ICSOL, intracranial space-occupying lesion; M, male; F, female; ARI, acute respiratory infection.

### Salmonella enterica.

CHRF0082 was a 4-month-old boy admitted with suspected meningitis, with fever and bulging fontanel. The CSF specimen tested in this study was collected 8 days after admission and contained 400 WBC/μl (80% polymorphonuclear neutrophils [PMNs]) and 450 μg/dl protein. The child was treated with ceftriaxone, netilmicin, and amoxicillin and discharged after 23 days with residual Bell’s palsy. mNGS identified S. enterica (Table S5), and retrospective investigation revealed that a CSF specimen was also collected the day of admission and that first specimen was culture positive for S. enterica. The child was healthy upon follow-up at 15 months of age.

### Mycobacterium tuberculosis.

CHRF0058 was a 13-year-old girl admitted after 30 days of fever, vomiting, and headache with neuroimaging observations of intracranial space-occupying lesions. Her CSF had 360 WBC/μl (80% PMNs) and 200 μg/dl protein. After 15 days of treatment with empirical ceftriaxone, meropenem, azithromycin, and acyclovir, the family left against medical advice. mNGS identified Mycobacterium tuberculosis (TB). Follow-up revealed that the child went to several health care facilities, where she was ultimately diagnosed with TB meningitis and initiated on antitubercular chemotherapy. The grandfather of the child lived in the same household and died from pulmonary TB 2 to 3 months before the onset of her symptoms. The child, after almost a year, remains bedridden with persistent neurocognitive impairment.

### Stenotrophomonas maltophilia.

CHRF0059 was a 4-month-old girl admitted for 10 days of fever, convulsion, and cough. She was treated at home with empirical cefixime and azithromycin before seeking care at Dhaka Shishu Hospital (DSH). Her CSF sample contained 120 WBC/μl (80% PMNs) and 300 μg/dl protein. She was treated with ceftriaxone, meropenem, vancomycin, and amikacin and discharged after 28 days. S. maltophilia was detected by mNGS (Table S5). This child was lost to follow-up.

### Bacillus cereus.

CHRF0070 was a 6-day-old boy admitted with fever, convulsion, lethargy, and yellow coloration of skin; the treating physicians provisionally diagnosed him with sepsis and neonatal jaundice. His CSF contained 12,000 WBC/μl (95% PMNs) and 500 μg/dl protein. He was discharged after 15 days following empirical treatment with ceftazidime and amikacin. mNGS identified B. cereus as the potential etiology. Follow-up at the age of 1 year revealed that the child required ventriculoperitoneal shunt placement for hydrocephalus. The child currently does not have any significant health problems.

### Mumps virus.

CHRF0036 was a 13-year-old boy admitted after 7 days of fever with irritability and headache. His CSF had 1,500 WBC/μl (60% PMNs) and 160 mg/dl protein. The patient was treated empirically with ceftriaxone for 10 days, after which the family left against medical advice. The logistic regression classifier failed to identify a potential pathogenic microbe. However, manual inspection of the data identified two reads that mapped mumps virus. This was further confirmed with validated qPCR of the original CSF sample to detect mumps and Sanger sequencing of the resultant amplicon. Mumps virus was not detected in any other samples, except for CHRF0011. During a follow-up conversation with the father, he reported that the child had had parotitis and fever preceding the headaches and that he had made a full recovery.

CHRF0011 was an 18-month-old boy admitted with fever and convulsion. His CSF revealed 100 WBC/μl (60% PMNs) and 60 μg/dl protein. He was treated with ceftriaxone and discharged after 9 days. mNGS identified mumps virus with sufficient read coverage to determine the genotype as G. At the age of 2.5 years, the child was healthy.

### Enterovirus B.

CHRF0010 was a 7-month-old boy admitted due to fever, convulsion, and lethargy. His CSF analysis unveiled 314 WBC/μl (80% PMNs) and 40 μg/dl protein. He was discharged after 6 days of empirical treatment with ceftriaxone. mNGS identified enterovirus B. Almost a year after the episode, the father reported that his child falls frequently.

### Chikungunya virus.

CHRF0071 was a 1-month-old girl admitted with fever, rash, convulsion, diarrhea, and lethargy. Her CSF contained 180 WBC/μl (80% PMNs) and 250 μg/dl protein. She was treated with ceftazidime and amikacin and discharged after 6 days. mNGS detected CHIKV with complete genome coverage. The child is currently healthy.

CHRF0094 was a 5-day-old girl admitted with fever, convulsion, and lethargy. CSF contained 1,000 WBC/μl (90% PMNs) and 220 μg/dl protein. She was treated with ceftazidime and amikacin, followed by meropenem, and was discharged after 10 days. mNGS detected CHIKV with complete genome coverage. The child was healthy during our follow up.

CHIRF0012 was an 86-month-old girl admitted with acute glomerulonephritis, fever, convulsion, abdominal distension, edema, lethargy, and generalized weakness. A lumbar puncture (LP) was performed after 36 days, presumably due to development of meningitis-like symptoms. The CSF contained 180 WBC/μl (60% PMNs) and 55 μg/dl protein. She was treated empirically with cefuroxime, metronidazole, ciprofloxacin, ceftazidime, and acyclovir. The child died after 45 days. mNGS identified CHIKV.

### Neuroinvasive CHIKV in Bangladesh.

**FIG 3:**
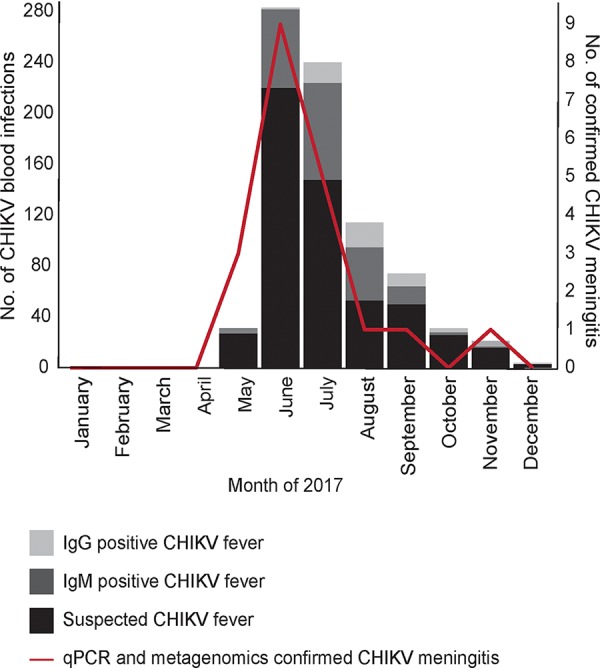
Chikungunya meningitis outbreak in Bangladesh. The CHIKV meningitis outbreak overlapped the CHIKV febrile illness outbreak. The months in 2017 show when the CHIKV-positive meningitis CSF samples were collected and when suspected febrile CHIKV cases sought care in the two largest pediatric hospitals of Bangladesh, Dhaka Shishu Hospital and Shishu Shasthya Foundation Hospital. The blood samples of suspected febrile CHIKV cases were detected by a specific diagnostic test for CHIKV-IgG and IgM (SD Biosensor, South Korea) as part of clinical care, and results were collected retrospectively from laboratory records.

All belonging to the East/Central/South African (ECSA) genotype, the three CHIKV genomes were >99% identical to each other and to the genome of the strain that caused a febrile outbreak in Dhaka in the summer of 2017. Two of the three children with CHIKV were admitted in June and July of 2017, the peak of the febrile outbreak. To determine if there were additional cases of meningitis admitted in DSH during that period, we performed CHIKV-specific qPCR on 472 idiopathic CSF specimens collected and stored in 2017 and identified 17 additional CHIKV cases. The dates of collection of the 20 CHIKV-positive CSF samples overlapped the dates when febrile CHIKV cases appeared in our hospitals (Fig. 3). Most of these cases originated in Dhaka city, where the febrile outbreak occurred (Fig. S2). The median age of these 20 CHIKV-positive patients was 8 months (range: 8 days to 96 months), and 35% were female. The mean CSF WBC count was 188/μl (range: 12 to 1,200 WBC/μl), and the mean PMNs were 48% (range: 10 to 90%). The average hospital length of stay was 11 days (range: 2 to 45 days), and the 30-day mortality rate was 0.05% (1/20).

10.1128/mBio.02877-19.4 FIG S2 Residential locations of all cases and CHIKV-positive meningitis cases. Download FIG S2, PDF file, 0.2 MB. Copyright © 2019 Saha et al. 2019 Saha et al. This content is distributed under the terms of the Creative Commons Attribution 4.0 International license.

**FIG 4:**
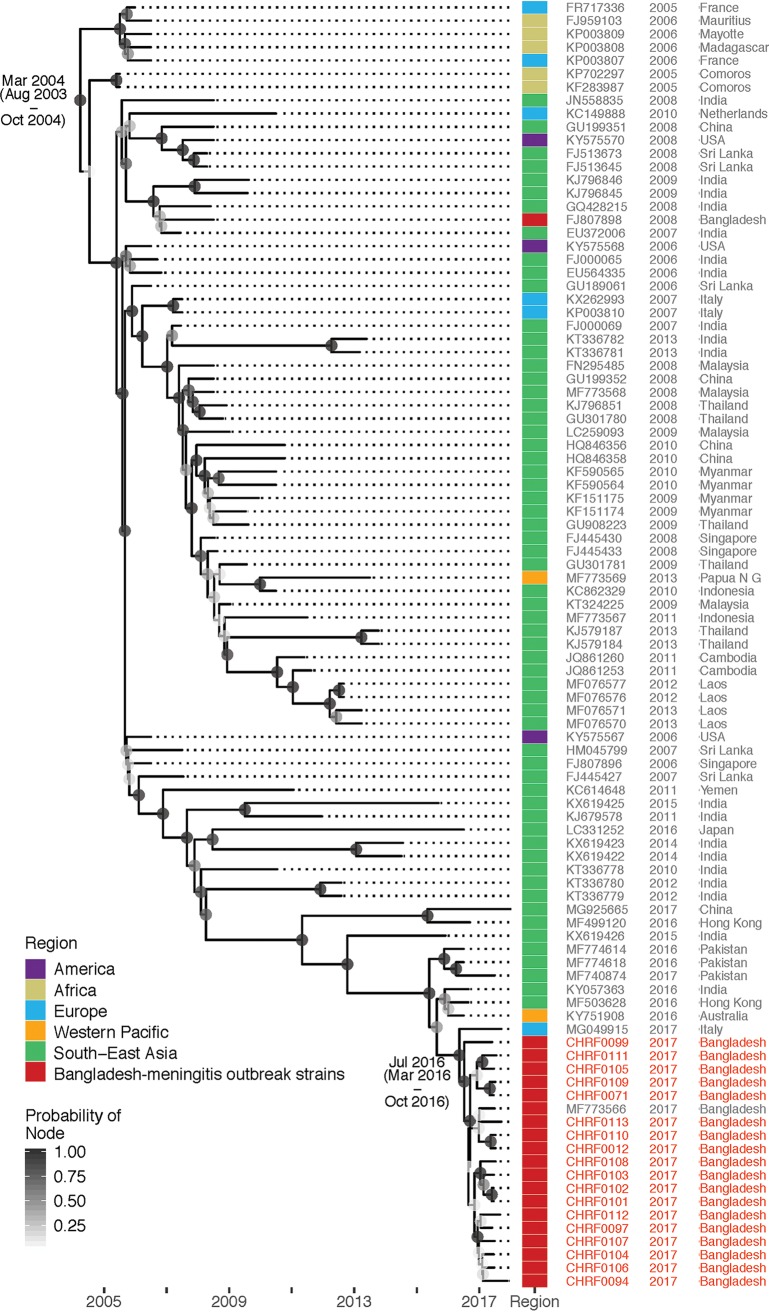
Genetic relationship of Bangladeshi CHIKV meningitis strain with strains that caused recent outbreak(s) in Bangladesh and elsewhere. All CHIKV genomes identified and assembled in this study were compared with selected CHIKV genomes available in NCBI data for time-resolved phylogenetics.

Subsequent mNGS of CSF from these 17 additional cases identified CHIKV RNA in all (Table S2). Comparison with other CHIKV genomes showed close relationship of the Bangladeshi strain with other strains that caused outbreaks in Asia in recent years, specifically to the one that caused an outbreak in Pakistan in 2016 (99.8% identity, Fig. 4).

## DISCUSSION

Prevention and effective and timely treatment of pediatric meningitis in LMICs is essential for achieving the United Nations’ Sustainable Development Goal 3 of ensuring healthy lives and promoting well-being for all at all ages. Obtaining a microbiological diagnosis can improve outcomes by informing targeted antimicrobial therapy for individual patients. On a population level, improved surveillance better informs region-specific treatment and prevention policy decisions and outbreak tracking. Here, we coupled unbiased RNA mNGS and machine learning with traditional diagnostics to bridge knowledge gaps and identify the etiologies of meningitis in Bangladesh. Metagenomic analysis using isolated RNA was chosen in order to detect pathogens that lack DNA intermediates, such as CHIKV and other RNA viruses. Furthermore, previous mNGS comparisons demonstrated a significant sensitivity advantage when using RNA input relative to DNA input, especially for challenging agents such as fungi (11).

For samples with known etiology previously determined by culture, qPCR, and/or antigen testing, mNGS correctly identified the pathogen in 25 of 36 (69%) samples; no false positives were detected. After excluding cases with low pathogen abundance as defined by a confirmatory qPCR *C_T_* of >32, mNGS identified 24 of 27 (89%) known infections. The decreased sensitivity of CSF mNGS for very-low-abundance pathogens has been previously reported (15). A potential pathogen was identified in 40% (10/25) of previously idiopathic meningitis cases. This included bacteria, mycobacteria, and viruses with established CNS pathogenicity that were likely not detected due to a variety of reasons, including prior antibiotic consumption, the lack of available clinical laboratory assays, and in the case of the CHIKV cases, lack of clinical suspicion for a newly emerging virus with underrecognized neuroinvasive potential.

Among potential bacterial pathogens, we identified S. enterica in one sample. Although that CSF specimen was culture negative, a culture obtained 1 week earlier grew S. enterica, suggesting that mNGS identified an infection even after antibiotic treatment cleared live pathogens. Bacillus cereus, detected in a 6-day-old neonate, is commonly recognized as a CNS pathogen in immunocompromised patients, including neonates, and at least 7 cases of neonatal B. cereus meningitis have been reported (16, 17). S. maltophilia was identified in a 4-month-old patient; this is a global emerging multidrug-resistant Gram-negative species of bacteria frequently associated with infections in young or immunocompromised patients (18). Mycobacterium tuberculosis, a well-known cause of severe and chronic meningitis in countries of TB endemicity (19), was detected from one patient. This finding was strengthened by the patient’s potential exposure in the household and a serendipitous subsequent diagnosis unrelated to this study. The time-consuming nature of culture and the low yield of smear-based detection make detection of TB meningitis notoriously challenging and suggest the potential value of mNGS for broad-range screening of diverse pathogens, including TB.

Among the viral pathogens detected in this study, mumps virus, an organism well associated with meningitis, was detected in two cases (20, 21). Enterovirus B, also a known cause of pediatric meningitis in South Asia, was detected in one case (22).

One of the most interesting findings in this study was the detection of CHIKV in three children, two of which cases occurred amid a 2017 CHIKV outbreak in Bangladesh. In recent years, there have been increasing reports of neurological complications associated with CHIKV infection (23, 24), but no neuroinvasive cases have been identified previously in Bangladesh, and no clinical testing for CHIKV in CSF was being performed in Bangladesh at the time. Our mNGS findings led us to retrospectively screen 472 CSF samples from DSH collected through 2017 with a CHIKV qPCR assay. This identified an additional 17 CHIKV cases, bringing the total to 20. The rate of detection of CHIKV meningitis was consistent with the rate of detection of CHIKV fever outbreak cases reported in Dhaka during the same time period. This study has enhanced the viral detection capabilities at the DSH laboratory, consistent with outbreak preparedness goals. Future studies will include a health care utilization survey in the catchment area surrounding the DSH to ascertain disease incidence (25), in addition to follow-up surveillance for neurodevelopmental assessment of the affected children.

The findings of this study should be considered within the context of several limitations. Importantly, CSF specimens were not collected and stored specifically for metagenomic analysis; this study used samples stored according to no stringent or consistent guidelines over a period of 6 years, which may have affected the quality of the RNA in the samples. Furthermore, this was a retrospective study, and thus, the timing of sample collection with respect to days of disease evolution and prior antibiotic exposure was uncontrolled and unknown in most cases. The quantity of pathogen material may be limiting or undetectable with time, which may contribute to the proportion of unresolved cases in this study. Future studies will include prospective surveillance following improved guidelines for sample collection, storage, processing, and sequencing and conducting mNGS on site, in real time. In this validation and application study, we used a high-output sequencing machine (Illumina NovaSeq) for sequencing. The high read count from many of the pathogens detected here suggest that lower-output machines (fewer reads) would be more than sufficient. However, in cases where the quantity of input RNA is extremely low, it may be challenging to automatically detect the pathogen, such as in the mumps virus case described here. Encouragingly, emerging methods for efficient host depletion (26) and pathogen enrichment (27) have shown promise for enhancing sensitivity of low-copy-number targets using sequencers with more modest output.

Our findings have opened several avenues for further exploration, discovery, and, in turn, evidence-based policy decisions for achieving Sustainable Development Goal 3. Unbiased metagenomic studies, guided by careful selection of positive and negative controls, can facilitate attribution of etiology to meningitis cases where traditional techniques have failed. Inclusion of machine learning techniques facilitates analysis of metagenomic data and complements methods independent of nucleic acid, such as direct antigen testing or serology. While we do not foresee administration of metagenomics for everyday diagnosis in LMICs in the near future, nor foresee mNGS replacing existing routine techniques in the near term, it is an excellent complementary tool that can be used in established surveillance platforms in regions of endemicity, in both outbreak and nonoutbreak situations. As shown in this study, CHIKV RNA was detected using metagenomics, and the outbreak of CHIKV meningitis was subsequently revealed by a low-cost qPCR technique guided by the findings of metagenomics. Ultimately, these improved patient- and population-level data can inform better health policy decisions, including but not limited to vaccine deployment, antibiotic stewardship, vector control, and pandemic preparedness.

## MATERIALS AND METHODS

### Study site and population.

All CSF samples used in this study were collected as part of the meningitis surveillance study supported by the WHO conducted in Dhaka Shishu Hospital (DSH). Children admitted at DSH were enrolled if they met WHO-defined inclusion criteria of meningitis and if a CSF specimen was collected (see Table S1 in the supplemental material) (28).

10.1128/mBio.02877-19.5 TABLE S1 WHO-defined clinical criteria used to enroll children in the meningitis surveillance in Dhaka Shishu Hospital, Bangladesh. Download Table S1, PDF file, 0.1 MB. Copyright © 2019 Saha et al. 2019 Saha et al. This content is distributed under the terms of the Creative Commons Attribution 4.0 International license.

Protocols were approved by the ethical review board of the Bangladesh Institute of Child Health. Samples were collected for routine clinical care, at the discretion of the attending physician, and informed consent was obtained from parents/caregivers.

### Laboratory methods and data collection.

CSF specimens were cultured (Fig. 1A) using standard procedures, and pneumococcal antigen was detected by immunochromatographic test (BinaxNow) (29–31). White blood cells (WBC) in the specimens were counted and differentiated into lymphocytes and polymorphonuclear neutrophils (PMNs). Culture-negative and pneumococcal-antigen-negative CSF specimens underwent latex agglutination and PCR testing for Haemophilus influenzae, pneumococcus, and meningococcus. Surplus CSF was stored at 4°C until it was transferred to −80°C, usually within 2 to 72 h. No stringent guidelines were followed, and time lag was not documented. Detection of Chikungunya virus (CHIKV) was conducted using qPCR with published primers on all CSF specimens stored and collected in 2017 (*n* = 472) (Table S2) (32).

10.1128/mBio.02877-19.6 TABLE S2 Detection of Chikungunya virus and orthogonal confirmation of probable pathogens through qPCR. Download Table S2, PDF file, 0.1 MB. Copyright © 2019 Saha et al. 2019 Saha et al. This content is distributed under the terms of the Creative Commons Attribution 4.0 International license.

### Sample selection.

Samples collected between 2012 and 2018 were selected, and clinical details of all selected samples are provided in Table S3. For positive controls, CSF specimens where an etiology could be successfully established through culture, antigen testing, and/or qPCR were chosen. For idiopathic samples, specimens were randomly chosen from a set that contained ≥20 WBC/μl (≥40% PMNs) (median: 314 WBC/μl) and ≥40 mg/dl protein (median: 220 mg/dl; normal range: 15 to 45 mg/dl).

10.1128/mBio.02877-19.7 TABLE S3 Case-based clinical and laboratory metadata of all cases included in this study (*n* = 115). Download Table S3, PDF file, 0.1 MB. Copyright © 2019 Saha et al. 2019 Saha et al. This content is distributed under the terms of the Creative Commons Attribution 4.0 International license.

Negative controls consisted of randomly chosen CSF specimens from patients in whom an alternate diagnosis was ultimately made, the child was discharged within 6 days of hospitalization, and CSF samples contained ≤6 WBC/μl (median: 0 WBC/μl) and ≤30 mg/dl protein (median: 20 mg/dl). This set also included environmental samples, which were nuclease-free water (Invitrogen, 10977-015) samples (*n* = 5) transferred into CSF collection tubes in the patient wards and treated and stored in the laboratory like CSF specimens in a blinded fashion. A “no-template” water control sample was included during RNA extraction.

### mNGS and confirmatory testing.

Total RNA was extracted from 100 μl of CSF, and mNGS libraries were prepared following published methods (14). External RNA Controls 103 Consortium collection (ERCC) (ThermoFisher, 4456740) spike-in controls were used in every sample as markers of potential library preparation errors and for input RNA mass calculation. Libraries were sequenced on a NovaSeq 6000 to generate 150-bp, paired-end sequences.

Pathogens were identified from the raw fastq files using the IDseq portal, a cloud-based, open-source bioinformatics platform designed for detection of microbes from metagenomic data (https://IDseq.net, v1.8 [Fig. S1 and Text S1]). Similar to published methods, potentially pathogenic microbes were distinguished from both ubiquitous environmental contaminants and commensal flora using a Z-score metric for each genus relative to a background distribution derived from the set of CSF specimens from nonmeningitis cases and water controls (10). Taxa with a Z-score less than 1 were removed from analysis. To further aid analysis, we employed a published logistic regression method to classify and assign potential etiological candidates in each sample (12). We retrained the model using the following features: RNA sequence reads per million (rpM), rank among all detected microbes within the sample, and a binary variable indicating whether the microbe has established pathogenicity (12, 33) (Table S4). Microbes with probability scores >0.2 were reported as potential pathogens. In cases where more than one potential pathogen was identified, only the top-scoring pathogen was considered. Based on the water controls, a minimum calculated RNA input threshold of 3.0 pg was required for pathogen prediction. The average RNA input of the set of noninfectious CSF samples was 1.6 pg (range: 0.9 to 2.8). Potential pathogens identified by mNGS were confirmed through PCR and Sanger sequencing (Table S2).

10.1128/mBio.02877-19.8 TABLE S4 List of microbes included in the logistic regression model as potential pathogens. Download Table S4, PDF file, 0.1 MB. Copyright © 2019 Saha et al. 2019 Saha et al. This content is distributed under the terms of the Creative Commons Attribution 4.0 International license.

10.1128/mBio.02877-19.9 TABLE S5 Case-based metagenomic data derived from all sequenced samples (*n* = 115). Download Table S5, PDF file, 0.1 MB. Copyright © 2019 Saha et al. 2019 Saha et al. This content is distributed under the terms of the Creative Commons Attribution 4.0 International license.

### Genome assembly, microbial typing, and phylogenetics.

For *de novo* assembly and annotation of draft genomes, we used the St. Petersburg genome assembler (SPAdes, v3.11.1) (34) and Geneious (v10.3.2) (35). Genotype assignments for viruses were identified using BLASTn, with the assembled genome sequence of the virus as query. We specifically compared the assembled CHIKV genomes with selected CHIKV genomes available in NCBI data for time-resolved phylogenetics (Text S2).

10.1128/mBio.02877-19.2 TEXT S2 Phylogenetic analysis of Chikungunya virus strains responsible for the meningitis outbreak in Bangladesh, 2017. Download Text S2, PDF file, 0.1 MB. Copyright © 2019 Saha et al. 2019 Saha et al. This content is distributed under the terms of the Creative Commons Attribution 4.0 International license.

### Clinical data collection and patient follow-up.

Clinical and demographic data were collected from electronically stored surveillance forms. The number and distribution of suspected and confirmed CHIKV febrile cases were collected from the microbiology laboratory records of Shishu Shasthya Foundation Hospital and DSH, the two largest pediatric hospitals of Bangladesh, serving the same catchment area. Clinical follow-up was conducted through telephone and/or home visits using structured questionnaires.

### Data availability.

All nonhuman sequence reads were deposited in the Sequence Read Archive, National Center for Biotechnology Information (NCBI), under BioProject no. PRJNA516582.

## References

[1] GBD 2017 Causes of Death Collaborators. 2018 Global, regional, and national age-sex-specific mortality for 282 causes of death in 195 countries and territories, 1980–2017: a systematic analysis for the Global Burden of Disease Study 2017. Lancet 392:1736–1788. doi:10.1016/S0140-6736(18)32203-7. 30496103 PMC6227606

[2] GBD 2017 Disease and Injury Incidence and Prevalence Collaborators. 2018 Global, regional, and national incidence, prevalence, and years lived with disability for 354 diseases and injuries for 195 countries and territories, 1990–2017: a systematic analysis for the Global Burden of Disease Study 2017. Lancet 392:1789–1858. doi:10.1016/S0140-6736(18)32279-7. 30496104 PMC6227754

[3] Brown JR, Bharucha T, Breuer J 2018 Encephalitis diagnosis using metagenomics: application of next generation sequencing for undiagnosed cases. J Infect 76:225–240. doi:10.1016/j.jinf.2017.12.014. 29305150 PMC7112567

[4] Ramakrishnan M, Ulland AJ, Steinhardt LC, Moïsi JC, Were F, Levine OS 2009 Sequelae due to bacterial meningitis among African children: a systematic literature review. BMC Med 7:47. doi:10.1186/1741-7015-7-47. 19751516 PMC2759956

[5] Granerod J, Tam CC, Crowcroft NS, Davies NWS, Borchert M, Thomas SL 2010 Challenge of the unknown: a systematic review of acute encephalitis in non-outbreak situations. Neurology 75:924–932. doi:10.1212/WNL.0b013e3181f11d65. 20820004

[6] Granerod J, Crowcroft NS 2007 The epidemiology of acute encephalitis. Neuropsychol Rehabil 17:406–428. doi:10.1080/09602010600989620. 17676528

[7] World Health Organization. 2011 Laboratory methods for the diagnosis of meningitis caused by Neisseria meningitidis, Streptococcus pneumoniae, and Haemophilus influenzae: WHO manual. World Health Organization, Geneva, Switzerland.

[8] Hasan AZ, Saha S, Saha SK, Sahakyan G, Grigoryan S, Mwenda JM, Antonio M, Knoll MD, Serhan F, Cohen AL 2018 Using pneumococcal and rotavirus surveillance in vaccine decision-making: a series of case studies in Bangladesh, Armenia and the Gambia. Vaccine 36:4939–4943. doi:10.1016/j.vaccine.2018.06.001. 30037484

[9] Glaser CA, Gilliam S, Schnurr D, Forghani B, Honarmand S, Khetsuriani N, Fischer M, Cossen CK, Anderson LJ 2003 In search of encephalitis etiologies: diagnostic challenges in the California Encephalitis Project, 1998–2000. Clin Infect Dis 36:731–742. doi:10.1086/367841. 12627357

[10] Wilson MR, O’Donovan BD, Gelfand JM, Sample HA, Chow FC, Betjemann JP, Shah MP, Richie MB, Gorman MP, Hajj-Ali RA, Calabrese LH, Zorn KC, Chow ED, Greenlee JE, Blum JH, Green G, Khan LM, Banerji D, Langelier C, Bryson-Cahn C, Harrington W, Lingappa JR, Shanbhag NM, Green AJ, Brew BJ, Soldatos A, Strnad L, Doernberg SB, Jay CA, Douglas V, Josephson SA, DeRisi JL 2018 Chronic meningitis investigated via metagenomic next-generation sequencing. JAMA Neurol 75:947–955. doi:10.1001/jamaneurol.2018.0463. 29710329 PMC5933460

[11] Zinter MS, Dvorak CC, Mayday MY, Iwanaga K, Ly NP, McGarry ME, Church GD, Faricy LE, Rowan CM, Hume JR 2018 Pulmonary metagenomic sequencing suggests missed infections in immunocompromised children. bioRxiv 291864. 10.1093/cid/ciy802 PMC6784263 30239621

[12] Langelier C, Kalantar KL, Moazed F, Wilson MR, Crawford E, Deiss T, Belzer A, Bolourchi S, Caldera S, Fung M, Jauregui A, Malcolm K, Lyden A, Khan L, Vessel K, Quan J, Zinter M, Chiu CY, Chow ED, Wilson J, Miller S, Matthay MA, Pollard KS, Christenson S, Calfee CS, DeRisi JL 2018 Integrating host response and unbiased microbe detection for lower respiratory tract infection diagnosis in critically ill adults. Proc Natl Acad Sci U S A 115:E12353–E12362. doi:10.1073/pnas.1809700115. 30482864 PMC6310811

[13] Loman NJ, Constantinidou C, Christner M, Rohde H, Chan JZ-M, Quick J, Weir JC, Quince C, Smith GP, Betley JR, Aepfelbacher M, Pallen MJ 2013 A culture-independent sequence-based metagenomics approach to the investigation of an outbreak of Shiga-toxigenic Escherichia coli O104:H4. JAMA 309:1502–1510. doi:10.1001/jama.2013.3231. 23571589

[14] Mayday MY, Khan LM, Chow ED, Zinter MS, DeRisi JL 2019 Miniaturization and optimization of 384-well compatible RNA sequencing library preparation. PLoS One 14:e0206194. doi:10.1371/journal.pone.0206194. 30629604 PMC6328170

[15] Miller S, Naccache SN, Samayoa E, Messacar K, Arevalo S, Federman S, Stryke D, Pham E, Fung B, Bolosky WJ, Ingebrigtsen D, Lorizio W, Paff SM, Leake JA, Pesano R, DeBiasi R, Dominguez S, Chiu CY 2019 Laboratory validation of a clinical metagenomic sequencing assay for pathogen detection in cerebrospinal fluid. Genome Res 29:831–842. doi:10.1101/gr.238170.118. 30992304 PMC6499319

[16] Tokieda K, Morikawa Y, Maeyama K, Mori K, Ikeda K 1999 Clinical manifestations of Bacillus cereus meningitis in newborn infants. J Paediatr Child Health 35:582–584. doi:10.1046/j.1440-1754.1999.00405.x. 10620178

[17] Gaur AH, Patrick CC, McCullers JA, Flynn PM, Pearson TA, Razzouk BI, Thompson SJ, Shenep JL 2001 Bacillus cereus bacteremia and meningitis in immunocompromised children. Clin Infect Dis 32:1456–1462. doi:10.1086/320154. 11317247

[18] Brooke JS 2012 Stenotrophomonas maltophilia: an emerging global opportunistic pathogen. Clin Microbiol Rev 25:2–41. doi:10.1128/CMR.00019-11. 22232370 PMC3255966

[19] Wilkinson RJ, Rohlwink U, Misra UK, Van Crevel R, Mai NTH, Dooley KE, Caws M, Figaji A, Savic R, Solomons R, Thwaites GE, Tuberculous Meningitis International Research Consortium. 2017 Tuberculous meningitis. Nat Rev Neurol 13:581–598. doi:10.1038/nrneurol.2017.120. 28884751

[20] Johnstone JA, Ross CAC, Dunn M 1972 Meningitis and encephalitis associated with mumps infection: a 10-year survey. Arch Dis Child 47:647–651. doi:10.1136/adc.47.254.647. 5046780 PMC1648314

[21] Bockelman C, Frawley TC, Long B, Koyfman A 2018 Mumps: an emergency medicine-focused update. J Emerg Med 54:207–214. doi:10.1016/j.jemermed.2017.08.037. 29110978

[22] Kumar A, Shukla D, Kumar R, Idris MZ, Misra UK, Dhole TN 2012 Molecular epidemiological study of enteroviruses associated with encephalitis in children from India. J Clin Microbiol 50:3509–3512. doi:10.1128/JCM.01483-12. 22895040 PMC3486256

[23] Mehta R, Gerardin P, de Brito CAA, Soares CN, Ferreira MLB, Solomon T 2018 The neurological complications of chikungunya virus: a systematic review. Rev Med Virol 28:e1978. doi:10.1002/rmv.1978. 29671914 PMC5969245

[24] Oliveira JRM, Gérardin P, Couderc T, Randrianaivo H, Fritel X, Lecuit M 2016 Chikungunya virus-associated encephalitis: a cohort study on La Réunion Island, 2005–2009. Neurology 86:2025–2026. doi:10.1212/WNL.0000000000002732. 27217467

[25] Luby SP, Saha S, Andrews JR 2015 Towards sustainable public health surveillance for enteric fever. Vaccine 33:C3–C7. doi:10.1016/j.vaccine.2015.02.054. 25912287

[26] Gu W, Crawford ED, O’Donovan BD, Wilson MR, Chow ED, Retallack H, DeRisi JL 2016 Depletion of abundant sequences by hybridization (DASH): using Cas9 to remove unwanted high-abundance species in sequencing libraries and molecular counting applications. Genome Biol 17:41. doi:10.1186/s13059-016-0904-5. 26944702 PMC4778327

[27] Quan J, Langelier C, Kuchta A, Batson J, Teyssier N, Lyden A, Caldera S, McGeever A, Dimitrov B, King R, Wilheim J, Murphy M, Ares LP, Travisano KA, Sit R, Amato R, Mumbengegwi DR, Smith JL, Bennett A, Gosling R, Mourani PM, Calfee CS, Neff NF, Chow ED, Kim PS, Greenhouse B, DeRisi JL, Crawford ED 2019 FLASH: a next-generation CRISPR diagnostic for multiplexed detection of antimicrobial resistance sequences. Nucleic Acids Res 47:e83. doi:10.1093/nar/gkz418. 31114866 PMC6698650

[28] Murray J, Agócs M, Serhan F, Singh S, Deloria-Knoll M, O’Brien K, Mwenda JM, Mihigo R, Oliveira L, Teleb N, Ahmed H, Wasley A, Videbaek D, Wijesinghe P, Thapa AB, Fox K, Paladin FJ, Hajjeh R, Schwartz S, Van Beneden C, Hyde T, Broome C, Cherian T, Centers for Disease Control and Prevention. 2014 Global invasive bacterial vaccine-preventable diseases surveillance. MMWR Morb Mortal Wkly Rep 63:1159–1162. 25503919 PMC4584539

[29] Moïsi JC, Saha SK, Falade AG, Njanpop‐Lafourcade B‐M, Oundo J, Zaidi AKM, Afroj S, Bakare RA, Buss JK, Lasi R, Mueller J, Odekanmi AA, Sangaré L, Scott JAG, Deloria Knoll M, Levine OS, Gessner BD 2009 Enhanced diagnosis of pneumococcal meningitis with use of the Binax NOW immunochromatographic test of Streptococcus pneumoniae antigen: a multisite study. Clin Infect Dis 48:S49–S56. doi:10.1086/596481. 19191619 PMC2863072

[30] Saha SK, Darmstadt GL, Yamanaka N, Billal DS, Nasreen T, Islam M, Hamer DH 2005 Rapid diagnosis of pneumococcal meningitis: implications for treatment and measuring disease burden. Pediatr Infect Dis J 24:1093–1098. doi:10.1097/01.inf.0000190030.75892.78. 16371872

[31] Saha SK, Naheed A, Arifeen SE, Islam M, Al‐Emran H, Amin R, Fatima K, Brooks WA, Breiman RF, Sack DA, Luby SP 2009 Surveillance for invasive Streptococcus pneumoniae disease among hospitalized children in Bangladesh: antimicrobial susceptibility and serotype distribution. Clin Infect Dis 48:S75–S81. doi:10.1086/596544. 19191622

[32] Lanciotti RS, Kosoy OL, Laven JJ, Panella AJ, Velez JO, Lambert AJ, Campbell GL 2007 Chikungunya virus in US travelers returning from India, 2006. Emerg Infect Dis 13:764–767. doi:10.3201/eid1305.070015. 17553261 PMC2738459

[33] Saha SK, Schrag SJ, El Arifeen S, Mullany LC, Shahidul Islam M, Shang N, Qazi SA, Zaidi AKM, Bhutta ZA, Bose A, Panigrahi P, Soofi SB, Connor NE, Mitra DK, Isaac R, Winchell JM, Arvay ML, Islam M, Shafiq Y, Nisar I, Baloch B, Kabir F, Ali M, Diaz MH, Satpathy R, Nanda P, Padhi BK, Parida S, Hotwani A, Hasanuzzaman M, Ahmed S, Belal Hossain M, Ariff S, Ahmed I, Ibne Moin SM, Mahmud A, Waller JL, Rafiqullah I, Quaiyum MA, Begum N, Balaji V, Halen J, Nawshad Uddin Ahmed ASM, Weber MW, Hamer DH, Hibberd PL, Sadeq-Ur Rahman Q, Mogan VR, Hossain T, McGee L, Anandan S, Liu A, Panigrahi K, Abraham AM, Baqui AH 2018 Causes and incidence of community-acquired serious infections among young children in south Asia (ANISA): an observational cohort study. Lancet 392:145–159. doi:10.1016/S0140-6736(18)31127-9. 30025808 PMC6053599

[34] Bankevich A, Nurk S, Antipov D, Gurevich AA, Dvorkin M, Kulikov AS, Lesin VM, Nikolenko SI, Pham S, Prjibelski AD, Pyshkin AV, Sirotkin AV, Vyahhi N, Tesler G, Alekseyev MA, Pevzner PA 2012 SPAdes: a new genome assembly algorithm and its applications to single-cell sequencing. J Comput Biol 19:455–477. doi:10.1089/cmb.2012.0021. 22506599 PMC3342519

[35] Kearse M, Moir R, Wilson A, Stones-Havas S, Cheung M, Sturrock S, Buxton S, Cooper A, Markowitz S, Duran C, Thierer T, Ashton B, Meintjes P, Drummond A 2012 Geneious Basic: an integrated and extendable desktop software platform for the organization and analysis of sequence data. Bioinformatics 28:1647–1649. doi:10.1093/bioinformatics/bts199. 22543367 PMC3371832

