# Supplementary material for: Unbiased Metagenomic Sequencing for Pediatric Meningitis in Bangladesh Reveals Neuroinvasive Chikungunya Virus Outbreak and Other Unrealized Pathogens
Source: mBio. 2019 Dec 17;10(6):e02877-19. doi: 10.1128/mBio.02877-19 (PMC6918088; doi:10.1128/mBio.02877-19)
Supplement: TEXT S1 [file mBio.02877-19-s0001.pdf]

Method S1. Bioinformatic analysis and pathogen identification

Microbial pathogens were identified from raw sequencing reads using the IDseq Portal (https://idseq.net), a cloud-based, open-source bioinformatics platform designed for detection of microbes from metagenomic data (eFigure 1). IDseq scripts and user instructions are available at https://github.com/chanzuckerberg/idseq-dag and the graphical user interface web application for sample upload is available at https://github.com/chanzuckerberg/idseq-web. IDseq is conceptually based on previously implemented platforms,(1–3) but is optimized for scalable Amazon Web Services (AWS) cloud deployment. Bioinformatics data processing jobs are carried out on demand as Docker containers using AWS Batch. Alignments to the National Center for Biotechnology Information (NCBI) database are executed on dedicated auto scaling groups (ASG) of Amazon Elastic Compute Cloud (EC2) instances, with the number of server instances varied with job load. Fast downloads of the NCBI database from the Amazon Simple Storage Service to each new server instance are enabled by the open-source tool s3mi (https://github.com/chanzuckerberg/s3mi). Initial alignment and removal of reads derived from the human genome is performed using the Spliced Transcripts Alignment to a Reference (STAR) algorithm.(4) Low-quality reads, duplicates, and low-complexity reads are then removed using the Paired-Read Iterative Contig Extension (PRICE) computational package,(5) the CD-HIT-DUP tool(6) and a filter based on the Lempel-Ziv-Welch (LZW) compression score, respectively. A second round of human read filtering is carried out using bowtie2(7) Remaining reads are queried against the most recent version of the NCBI nucleotide (NT) and non-redundant (NR) protein databases (updated monthly) using GSNAPL and RAPSearch2 respectively.(8, 9) Reads matching GenBank records in the superphylum Deuterostomia are removed, given the high likelihood that such residual reads are of human origin. The relative abundance of microbial taxa is calculated based on reads per million (rpM) mapped at the genus level. An overview of this pipeline is represented in eFigure 1.

References:

1. Yozwiak NL, Skewes-Cox P, Stenglein MD, Balmaseda A, Harris E, DeRisi JL. 2012. Virus identification in unknown tropical febrile illness cases using deep sequencing. PLoS Negl Trop Dis 6:e1485
2. Wilson MR, O'Donovan BD, Gelfand JM, Sample HA, Chow FC, Betjemann JP, Shah MP, Richie MB, Gorman MP, Hajj-Ali RA, Calabrese LH, Zorn KC, Chow ED, Greenlee JE, Blum JH, Green G, Khan LM, Banerji D, Langelier C, Bryson-Cahn C, Harrington W, Lingappa JR, Shanbhag NM, Green AJ, Brew BJ, Soldatos A, Strnad L, Doernberg SB, Jay CA, Douglas V, Josephson SA, DeRisi JL. 2018. Chronic Meningitis Investigated via Metagenomic Next-Generation Sequencing. JAMA Neurol 75:947-955.
3. Naccache SN, Federman S, Veeraraghavan N, Zaharia M, Lee D, Samayoa E, Bouquet J, Greninger AL, Luk KC, Enge B, Wadford DA, Messenger SL, Genrich GL, Pellegrino K, Grard G, Leroy E, Schneider BS, Fair JN, Martínez MA, Isa P, Crump JA, DeRisi JL, Sittler T, Hackett J, Miller S, Chiu CY. 2014. A cloud-compatible bioinformatics pipeline for ultrarapid pathogen identification from next-generation sequencing of clinical samples. Genome Res 24:1180–1192.
4. Dobin A, Davis CA, Schlesinger F, Drenkow J, Zaleski C, Jha S, Batut P, Chaisson M, Gingeras TR. 2013. STAR: Ultrafast universal RNA-seq aligner. Bioinformatics 29:15–21.
5. Ruby JG, Bellare P, DeRisi JL. 2013. PRICE: software for the targeted assembly of components of (meta)genomic sequence data. G3 3:865–880.
6. Li W, Godzik A. 2006. Cd-hit: A fast program for clustering and comparing large sets of protein or nucleotide sequences. Bioinformatics 22:1658–1659.
7. Langmead B, Salzberg SL. 2012. Fast gapped-read alignment with Bowtie 2. Nat Methods 9:357–359.
8. Wu TD, Nacu S. 2010. Fast and SNP-tolerant detection of complex variants and splicing in short reads. Bioinformatics 26:873–881.
9. Ye Y, Choi J-H, Tang H. 2011. RAPSearch: a fast protein similarity search tool for short reads. BMC Bioinformatics 12:159.
