# Supplementary material for: Unbiased Metagenomic Sequencing for Pediatric Meningitis in Bangladesh Reveals Neuroinvasive Chikungunya Virus Outbreak and Other Unrealized Pathogens
Source: mBio. 2019 Dec 17;10(6):e02877-19. doi: 10.1128/mBio.02877-19 (PMC6918088; doi:10.1128/mBio.02877-19)

Figure S1. **Schematic representation of the bioinformatic approach in IDSEQ for pathogen identification.**

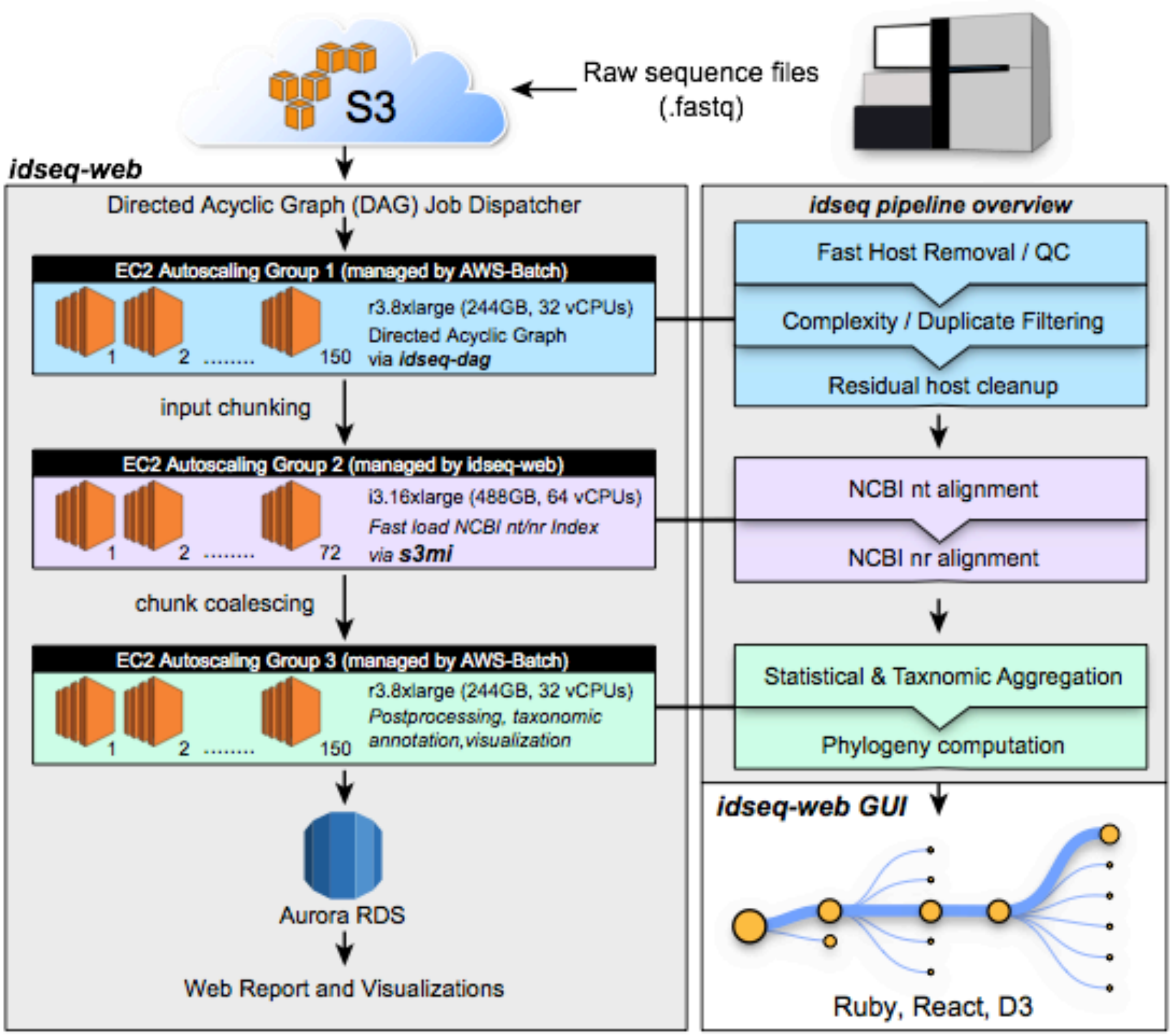

Supplement: FIG S1 [file mBio.02877-19-sf001.pdf]
