# Supplementary material for: Unbiased Metagenomic Sequencing for Pediatric Meningitis in Bangladesh Reveals Neuroinvasive Chikungunya Virus Outbreak and Other Unrealized Pathogens
Source: mBio. 2019 Dec 17;10(6):e02877-19. doi: 10.1128/mBio.02877-19 (PMC6918088; doi:10.1128/mBio.02877-19)

Figure S2. **Residential locations of all selected cases and CHIKV-positive meningitis cases.** A. The map of Bangladesh depicting national distribution of all cases. B. Magnified map of Dhaka division from where majority of cases arrived. C The map of Bangladesh depicting national distribution of all CHIKV-positive meningitis cases during the outbreak of 2017. D. Magnified map of Dhaka division from where almost of CHIKV-meningitis cases arrived from during the outbreak in 2017.

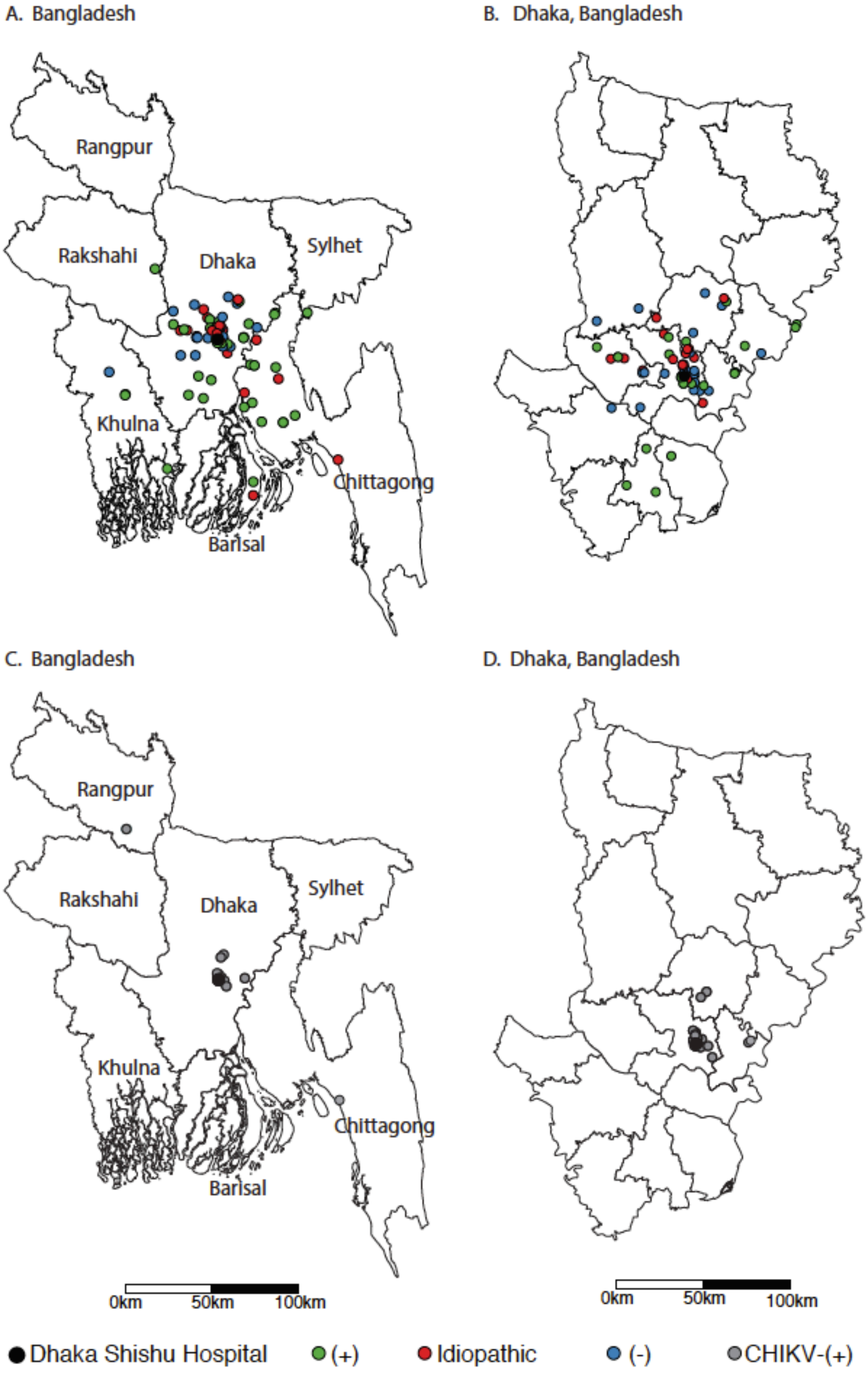

Supplement: FIG S2 [file mBio.02877-19-sf002.pdf]
