# Supplementary material for: Unbiased Metagenomic Sequencing for Pediatric Meningitis in Bangladesh Reveals Neuroinvasive Chikungunya Virus Outbreak and Other Unrealized Pathogens
Source: mBio. 2019 Dec 17;10(6):e02877-19. doi: 10.1128/mBio.02877-19 (PMC6918088; doi:10.1128/mBio.02877-19)
Supplement: TABLE S1 [file mBio.02877-19-st001.pdf]

Table S1. **WHO-defined clinical criteria used to enroll children in the meningitis surveillance in Dhaka Shishu Hospital, Bangladesh.**

WHO case definition	Inclusion criteria
Meningitis	Any child aged 0-59 months hospitalized with sudden onset of fever >100.4 °F and one of the following signs:<br>• Stiff neck<br>• Altered or reduced level of consciousness<br>• Bulging fontanel (if < 12 months of age)<br>• Prostration/lethargy<br>• Convulsions<br>• Toxic appearance<br>• Petechial or purpuric rash<br>• Poor Sucking<br>• Irritability (>2 months)<br>Or any child aged 0-59 months hospitalized with a clinical diagnosis of meningitis.
