# Supplementary material for: Unbiased Metagenomic Sequencing for Pediatric Meningitis in Bangladesh Reveals Neuroinvasive Chikungunya Virus Outbreak and Other Unrealized Pathogens
Source: mBio. 2019 Dec 17;10(6):e02877-19. doi: 10.1128/mBio.02877-19 (PMC6918088; doi:10.1128/mBio.02877-19)
Supplement: TABLE S2 [file mBio.02877-19-st002.pdf]

Table S2. **Detection of Chikungunya virus and orthogonal confirmation of probable pathogens through qPCR.** For CHIKV confirmation, we used qScript XLT 1-Step RT-qPCR ToughMix Low-ROX (Cat. No. 95134-100). For the remaining confirmations, we used Quantabio PerfeCTa qPCR ToughMix, Low Rox (Cat. No. 95114-012). For reverse transcription, we used Quantabio qScript cDNA Super Mix (Cat. No. 95048-100): 25°C for 5 min, 42°C for 30 min, 85°C for 5 min.

Sample ID	Pathogen	Template	Primers	Probes	qPCR conditions
CHRF_0012, CHRF_0071, CHRF_0094	CHIKV	Whole CSF*	F:5'AAAGGGCAAACTCAGCTTCAC; R: 5'GCCTGGGCTCATCGTTATTC	FAM-CACCGACGGCGAGACCGACTTT-BHQ1	50°C for 10 min, 95°C for 1 min, 45 cycles of 95°C for 15 s, 60°C for 1 min.
CHRF_0010	Enterovirus B	CSF-RNA	F:5'CCTGAATGCGGCTAATCC; R:5'TTGTCACCATWAGCAGYCA	FAM-CCGACTACTTTGGGWGTCCGTGT-BHQ1	95°C for 30 s, 40 cycles: 95°C for 15 s, 60°C for 30 s
CHRF_0011, CHRF_0036	Mumps	CSF-RNA	F:5'GTATGACAGCGTACGACCAACCT; R:5'GCGACCTTGCTGCTGGTATT 3'	FAM-CCGGGTCTGCTGATCGGCGAT-BHQ	95°C for 30 s, 40 cycles: 95°C for 5 s, 60°C for 15 s, 70°C for 10 s
CHRF_0059	*S. maltophilia*	CSF-RNA	F:5'GCCGAAAGCCCAAGGTTT; R:5'CGACTTTCGTCCTCGCCTTA 3'	FAM-CGCAACGTTAATCGGCGCAGG-TAMRA	95°C for 30 s, 40 cycles: 95°C for 5 s, 60°C for 15 s, 70°C for 10 s
CHRF_0082	*S. enterica*	Whole CSF	F:5'CTCACCAGGAGATTACAACATGG; R:5'AGCTCAGACCAAAAGTGACCATC	FAM-CACCGACGGCGAGACCGACTTT-BHQ1	95°C for 30 s, 40 cycles: 95°C for 15 s, 60°C for 30 s
CHRF_0058	*M. tuberculosis*	Whole CSF	F:5'GTCGAACGGCTGATGACCAAACT; R:5'TCCGAAGCGGCGCTGGACGA; F1:5'AACGGCTGATGACCAAACTC 3'; R1:5'AAGCGGCGCTGGACGAGATC	Probe1: FAM-ACCACGATCGCTGATCCGGCCACA-BHQ1; Probe2: FAM-ACCCGCGGCAAAGCCCGCAGG-BHQ1	95°C for 10 min, 95°C 10 min; 10 cycles: 95°C 15 s, 65°C 30 s; 40 cycles: 95°C 15s, 60°C 30 s
CHRF_0070	*B. cereus*	Whole CSF	F:5'ACTGTATTAGAAGGGCGCGG; R:5'TAAAGCCACCGCCAAGTACA 3'	N/A	95°C for 10 min, 95°C for 30 s , 40 cycles: 95°C for 5 s, 57°C for 15 s, 70°C for 10 s
