# Supplementary material for: Unbiased Metagenomic Sequencing for Pediatric Meningitis in Bangladesh Reveals Neuroinvasive Chikungunya Virus Outbreak and Other Unrealized Pathogens
Source: mBio. 2019 Dec 17;10(6):e02877-19. doi: 10.1128/mBio.02877-19 (PMC6918088; doi:10.1128/mBio.02877-19)
Supplement: TABLE S3 [file mBio.02877-19-st003.pdf]

Table S3. Case-based clinical and laboratory metadata of all cases included in this study (n=115).

Sample ID	DSH result	Age (m)	CSF collection date	WBC/ul (PMN%)/ Protein mg/dl	Hospital duration/ outcome	Organism detected in DSH	qPCR Ct	Provisional/ Final Diagnosis
CHRF0052	Culture (+)	f/3	14-Nov-17	6000 (90)/600	21/Dis	*E. coli*	-	Sepsis/Hydrocephalus
CHRF0064	Culture (+)	m/81	6-Jun-16	24000 (95)/1000	12/Dis	*S. pneumoniae*	-	Meningoencephalitis/ Meningitis
CHRF0076	Culture (+)	m/6	20-Feb-15	860 (90)/300	1/LAMA	*S. pneumoniae*	-	Meningitis/Meningitis
CHRF0088	Culture (+)	f/0	25-Sep-16	750 (90)/500	32/Dis	*Flavobacterium sp.*	-	Meningitis/Meningitis
CHRF0005	Culture (+)	m/0	5-Jun-16	3500 (90)/700	23/Dis	*Enterobacter sp.*	-	perinatal asphyxia/Meningitis
CHRF0017	Culture (+)	m/132	13-Mar-16	2600 (90)/150	24/Dis	*E. coli*	-	Meningitis/Meningitis
CHRF0029	Culture (+)	f/0	10-Jan-16	280 (85)/300	38/LAMA	*Flavobacterium sp.*	-	perinatal asphyxia /perinatal asphyxia
CHRF0041	Culture (+)	m/6	03-Nov-12	250 (50)/300	76/Dis	*K. pneumoniae*	-	Meningitis/Hydrocephalus
CHRF0001	Antigen, PCR (+)	m/0	16-May-17	250 (80)/300	15/Dis	*S. pneumoniae*	32.9	Meningitis/Meningitis
CHRF0013	Antigen, PCR (+)	m/4	7-Mar-17	74 (25)/60	13/Dis	*S. pneumoniae*	36	Meningitis/Meningitis
CHRF0025	Antigen, PCR (+)	f/3	19-Mar-17	52 (10)/200	15/Dis	*S. pneumoniae*	27.2	Meningitis/Meningitis
CHRF0037	Antigen, PCR (+)	m/60	18-Feb-17	5000 (90)/250	15/Dis	*S. pneumoniae*	28.7	Meningoencephalitis/ Meningitis
CHRF0049	Antigen, PCR (+)	100	5-Sep-17	2000 (80)/800	14/Dis	*S. pneumoniae*	26.5	Meningitis/Meningitis
CHRF0061	Antigen, PCR (+)	f/4	10-Apr-17	32000 (90)/300	26/Dis	*S. pneumoniae*	21.5	Meningitis/Pneumonia
CHRF0073	Antigen, PCR (+)	m/1	10-Apr-17	4000 (95)/700	7/Died	*S. pneumoniae*	23.4	Meningitis/Meningitis
CHRF0085	Antigen, PCR (+)	f/4	28-Mar-17	3200 (85)/500	26/Dis	*S. pneumoniae*	27.1	Meningitis/Meningitis
CHRF0002	Antigen, PCR (+)	m/3	27-Feb-17	80 (60)/300	7/LAMA	*S. pneumoniae*	19.2	Meningitis/Meningitis
CHRF0014	Antigen, PCR (+)	m/7	26-Feb-17	10000 (90)/400	5/LAMA	*S. pneumoniae*	27.6	Meningitis/Meningitis
CHRF0026	Antigen, PCR (+)	m/108	30-Aug-17	700 (90)/300	9/Died	*S. pneumoniae*	19	NS/NS
CHRF0038	Antigen, PCR (+)	m/79	19-Mar-16	4800 (80)/300	10/Dis	*S. pneumoniae*	29.7	Meningitis/Meningitis
CHRF0050	Antigen, PCR (+)	f/6	16-Mar-16	140 (70)/150	2/Dis	*S. pneumoniae*	31.8	Meningitis/Meningitis
CHRF0062	Antigen, PCR (+)	f/9	7-May-16	2500 (90)/250	12/Dis	*S. pneumoniae*	23.8	Meningitis/Meningitis
CHRF0074	Antigen, PCR (+)	f/85	27-Jun-16	3000 (85)/350	18/Dis	*S. pneumoniae*	27.7	Meningitis/Meningitis
CHRF0086	Antigen, PCR (+)	m/6	14-Dec-15	550 (90)/300	14/Dis	*S. pneumoniae*	26.2	Meningitis/Meningitis
CHRF0003	Antigen, PCR (+)	m/71	18-Jan-15	9600 (90)/220	14/Dis	*S. pneumoniae*	16.3	ICSOL/Meningitis
CHRF0015	Antigen (+)	m/78	22-Mar-15	80 (10)/15	18/Dis	*S. pneumoniae*	NA	NS/NS
CHRF0027	Antigen, PCR (+)	m/1	27-Sep-17	1300 (80)/250	15/Dis	*H. influenzae*	28.4	Meningitis/Meningitis

Sample ID	DSH result	Age (m)	CSF collection date	WBC/ul (PMN%)/ Protein mg/dl	Hospital duration/ outcome	Organism detected in DSH	qPCR Ct	Provisional/ Final Diagnosis
CHRF0039	Antigen, PCR (+)	f/4	4-Sep-16	950 (85)/70	9/Dis	*S. pneumoniae*	34.1	Meningitis/Meningitis
CHRF0051	Antigen, PCR (+)	m/3	14-May-16	6500 (90)/200	12/Dis	*S. pneumoniae*	21.3	Meningitis/Meningitis
CHRF0063	Antigen, PCR (+)	f/40	22-Jul-16	2400 (90)/200	9/Dis	*N. meningitidis*	33	Meningitis/Meningitis
CHRF0075	Antigen, PCR (+)	m/72	16-Feb-15	3400 (90)/200	27/Dis	*S. pneumoniae*	29.3	Meningoencephalitis/ Meningoencephalitis
CHRF0087	Antigen, PCR (+)	f/82	27-Nov-17	1600 (90)/80	28/Dis	*S. pneumoniae*	31.7	Meningoencephalitis/ Meningitis
CHRF0004	Antigen, PCR (+)	m/61	9-Sep-17	220 (10)/100	14/Dis	*S. pneumoniae*	35.5	Meningitis/Meningitis
CHRF0016	Antigen (+)	f/4	5-Oct-17	10 (60)/150	32/Dis	*S. pneumoniae*	NA	Encephalitis/Meningitis
CHRF0028	Antigen, PCR (+)	m/55	30-Oct-17	28 (30)/100	8/Dis	*S. pneumoniae*	33.2	Meningitis/Meningitis
CHRF0040	Antigen, PCR (+)	m/9	18-Dec-17	280 (15)/160	12/Dis	*S. pneumoniae*	36	Pneumonia
CHRF0053	(-)	f/12	23-Jul-14	0 (0)/15	3/Dis	-	-	UTI/AGE
CHRF0065	(-)	m/8	24-Jul-14	0 (0)/20	2/Dis	-	-	AFC/AFC
CHRF0077	(-)	m/5	19-Sep-14	0 (0)/25	4/Dis	-	-	AFC/AFC
CHRF0089	(-)	f/12	21-Sep-14	0 (0)/30	3/Dis	-	-	AFC/AFC
CHRF0006	(-)	f/5	21-Sep-14	0 (0)/15	4/Dis	-	-	Meningitis/ARI
CHRF0018	(-)	f/24	24-Sep-14	0 (0)/15	2/Dis	-	-	Meningitis/AFC
CHRF0030	(-)	m/14	29-Sep-14	0 (0)/15	3/Dis	-	-	AFC/AFC
CHRF0042	(-)	m/0	12-Oct-14	0 (0)/20	4/Dis	-	-	Perinatal asphyxia/ Perinatal asphyxia
CHRF0054	(-)	m/12	30-Oct-14	0 (0)/25	3/Dis	-	-	Meningitis/AFC
CHRF0066	(-)	m/16	21-Nov-14	0 (0)/25	3/Dis	-	-	AFC/AFC
CHRF0078	(-)	m/14	16-Jan-15	0 (0)/20	4/Dis	-	-	AFC/AFC
CHRF0090	(-)	m/12	22-Jan-15	0 (0)/15	4/Dis	-	-	AFC/AFC
CHRF0007	(-)	f/8	29-Jan-15	0 (0)/20	4/Dis	-	-	Meningitis/AFC
CHRF0019	(-)	f/12	1-Feb-15	0 (0)/20	4/Dis	-	-	Meningitis/AFC
CHRF0031	(-)	m/9	2-Feb-15	0 (0)/15	3/Dis	-	-	Down's syndrome/AFC
CHRF0043	(-)	f/5	5-Feb-15	0 (0)/20	2/Dis	-	-	AFC/AFC
CHRF0055	(-)	m/43	3-Feb-18	4 (0)/20	3/Dis	-	-	AFC/AFC
CHRF0067	(-)	f/28	3-Feb-18	2 (0)/20	3/Dis	-	-	AFC/AFC
CHRF0079	(-)	f/8	5-Feb-18	2 (0)/20	5/Dis	-	-	AFC/AFC
CHRF0091	(-)	m/17	10-Feb-18	2 (0)/20	3/Dis	-	-	AFC/AFC
CHRF0008	(-)	m/15	11-Feb-18	2 (0)/15	3/Dis	-	-	AFC/AFC
CHRF0020	(-)	m/6	11-Feb-18	2 (0)/20	3/Dis	-	-	Meningitis/AFC

Sample ID	DSH result	Age (m)	CSF collectio n date	WBC/ul (PMN%)/ Protein mg/dl	Hospital duration/ outcome	Organism detected in DSH	qPC R Ct	Provisional/ Final Diagnosis
CHRF0032	(-)	m/6	15-Feb-18	4 (0)/20	3/Dis	-	-	Pseudotumor cerebri/Pseudotumor cerebri
CHRF0044	(-)	f/27	15-Feb-18	2 (0)/20	3/Dis	-	-	Pseudotumor cerebri
CHRF0056	(-)	f/18	20-Feb-18	2 (0)/20	6/Dis	-	-	Seizure disorder/AFC
CHRF0068	(-)	m/8	17-Feb-18	0 (0)/20	2/Dis	-	-	Meningitis/AFC
CHRF0080	(-)	f/14	18-Feb-18	4 (0)/20	4/Dis	-	-	AFC/AFC
CHRF0092	(-)	m/11	19-Feb-18	6 (0)/25	3/Dis	-	-	AFC/AFC
CHRF0009	(-)	m/14	19-Feb-18	4 (0)/25	5/Dis	-	-	AFC/AFC
CHRF0021	water	f/8	28-Feb-18	2 (0)/25	4/Dis	-	-	AFC/AFC
CHRF0033	water	-	4-Mar-18	-	-	-	-	-
CHRF0045	water	-	4-Mar-18	-	-	-	-	-
CHRF0057	water	-	4-Mar-18	-	-	-	-	-
CHRF0069	water	-	4-Mar-18	-	-	-	-	-
CHRF0096	water	-	4-Mar-18	-	-	-	-	-
CHRF0000	water	-	-	-	-	-	-	-
CHRF0081	Idiopathic	m/72	5-Feb-18	1100 (40)/350	/LAMA	-	-	Meningitis/Tubercula r meningitis
CHRF0093	Idiopathic	m/13	22-Feb-18	20 (80)/80	11/Died	-	-	Congenital heart disease/Dextrocardia
CHRF0010	Idiopathic	m/7	4-Jan-18	314 (80)/40	6/Dis	-	-	Meningitis/Meningiti s
CHRF0022	Idiopathic	m/3	8-Jan-18	460 (80)/300	9/LAMA	-	-	Meningitis/Meningiti s
CHRF0034	Idiopathic	f/23	22-Jan-18	70 (70)/150	11/Dis	-	-	Meningitis/Meningiti s
CHRF0046	Idiopathic	f/1	28-Jan-18	160 (80)/70	4/LAMA	-	-	Pneumonia/Pneumoni a
CHRF0058	Idiopathic	f/160	27-Dec-04	360 (80)/200	15/LAMA	-	-	ICSOL/ICSOL
CHRF0070	Idiopathic	m/0	14-Dec-17	12000 (95)/500	15/Dis	-	-	Sepsis/Sepsis
CHRF0082	Idiopathic	m/4	9-Dec-17	90 (70)/600	23/Dis	-	-	Meningitis/Meningiti s
CHRF0094	Idiopathic	f/0	22-Nov-17	1000 (90)/220	10/Dis	-	-	Meningitis/Meningiti s
CHRF0011	Idiopathic	m/18	5-Nov-17	100 (60)/60	9/Dis	-	-	Meningitis/Meningiti s
CHRF0023	Idiopathic	m/4	18-Sep-17	74 (70)/100	8/Dis	-	-	Meningitis/Meningiti s
CHRF0035	Idiopathic	f/21	13-Sep-17	600 (90)/700	56/Died	-	-	ASS/ASS
CHRF0047	Idiopathic	m/16	5-Sep-17	2600 (90)/400	5/Died	-	-	ARI/Meningoenceph alitis
CHRF0059	Idiopathic	f/4	20-Aug-17	120 (80)/300	28/Dis	-	-	Meningitis/Meningiti s
CHRF0071	Idiopathic	f/1	17-Jun-17	180 (80)/250	6/Dis	-	-	Meningitis/Meningiti s
CHRF0083	Idiopathic	m/7	12-Aug-17	5600 (80)/1000	10/Died	-	-	Hydrocephalus/Pneu monia

Sample ID	DSH result	Age (m)	CSF collection date	WBC/ul (PMN%)/ Protein mg/dl	Hospital duration/ outcome	Organism detected in DSH	qPCR Ct	Provisional/ Final Diagnosis
CHRF0095	Idiopathic	m/98	16-Jul-17	90 (70)/60	33/LAMA	-	-	Meningoencephalitis/ TB meningitis
CHRF0012	Idiopathic	f/86	11-Jul-17	180 (60)/55	45/Died	-	-	AGN/Meningoencephalitis
CHRF0024	Idiopathic	f/96	25-Apr-17	460 (60)/200	8/LAMA	-	-	Meningitis/Meningitis
CHRF0036	Idiopathic	m/156	23-Apr-17	1500 (60)/160	10/LAMA	-	-	Meningitis/Meningitis
CHRF0048	Idiopathic	m/2	6-Jun-17	3800 (80)/400	35/Dis	-	-	Meningitis/Meningitis
CHRF0060	Idiopathic	m/4	4-Jun-17	84 (70)/150	11/LAMA	-	-	Meningitis/Meningitis
CHRF0072	Idiopathic	f/3	25-May-17	900 (60)/300	44/Dis	-	-	Meningitis/Meningitis
CHRF0084	Idiopathic	f/4	20-May-17	70 (60)/250	51/Died	-	-	Meningitis/Hydrocephalus
CHRF0097	PCR (+)	f/1	31-May-17	1200 (90)/250	12/Dis	CHIKV	36.0	Septicaemia/Meningitis
CHRF0098	PCR (+)	m/96	31-May-17	26 (10)/80	11/Dis	CHIKV	38.5	Encephalitis/Meningitis
CHRF0099	PCR (+)	f/11	31-May-17	20 (20)/30	4/Dis	CHIKV	35.0	AFC/Meningitis
CHRF0100	PCR (+)	m/22	14-Jun-17	60 (25)/70	2/Dis	CHIKV	37.7	AFC/AFC
CHRF0101	PCR (+)	m/4	17-Jun-17	160 (70)/110	8/Dis	CHIKV	31.3	AFC/Meningitis
CHRF0102	PCR (+)	m/3	17-Jun-17	220 (80)/250	15/Dis	CHIKV	35.4	AFC/Meningitis
CHRF0103	PCR (+)	m/93	19-Jun-17	20 (10)/40	4/Dis	CHIKV	33.7	Meningitis/AFC
CHRF0104	PCR (+)	m/11	19-Jun-17	20 (10)/30	4/Dis	CHIKV	37.9	Meningitis/AFC
CHRF0105	PCR (+)	f/14	21-Jun-17	32 (20)/35	2/LAMA	CHIKV	31.5	AFC
CHRF0106	PCR (+)	m/20	24-Jun-17	120 (90)/70	7/Dis	CHIKV	35.1	Meningitis/AFC
CHRF0107	PCR (+)	m/17	29-Jun-17	12 (10)/30	3/Dis	CHIKV	34.7	Meningitis/AFC
CHRF0108	PCR (+)	f/8	10-Jul-17	120 (30)/35	9/Dis	CHIKV	35.8	AFC/Meningitis
CHRF0109	PCR (+)	m/2	8-Jul-17	24 (70)/50	28/Dis	CHIKV	33.8	Pneumonia/Pneumonia
CHRF0110	PCR (+)	m/0	10-Jul-17	60 (90)/150	16/Dis	CHIKV	30.3	Sepsis/Meningitis
CHRF0111	PCR (+)	m/8	18-Jul-17	42 (20)/35	7/Dis	CHIKV	34.3	AFC/Meningitis
CHRF0112	PCR (+)	m/7	31-Aug-17	180 (10)/50	12/Dis	CHIKV	29.3	Meningitis/Meningitis
CHRF0113	PCR (+)	m/1	18-Sep-17	90 (70)/150	17/Dis	CHIKV	32.7	Meningitis/Meningitis
CHRF0114	Negative - Water	-	-	-	-	-	-	-

ICSOL: Intracranial; UTI: Urinary tract infection; ARI: Acute respiratory tract infection; AGN: acute glomerulonephritis; AGE: acute gastroenteritis; AFC: Acute febrile convulsion; ASS: Acute stroke syndrome; NS: Nephrotic syndrome; LAMA: Left against medical advice; NA: the data are not available or applicable
