# Supplementary material for: Unbiased Metagenomic Sequencing for Pediatric Meningitis in Bangladesh Reveals Neuroinvasive Chikungunya Virus Outbreak and Other Unrealized Pathogens
Source: mBio. 2019 Dec 17;10(6):e02877-19. doi: 10.1128/mBio.02877-19 (PMC6918088; doi:10.1128/mBio.02877-19)
Supplement: TABLE S4 [file mBio.02877-19-st004.pdf]

Table S4. **List of microbes included in the logistic regression model as potential pathogens.**

**Bacteria**	**Viruses**
Escherichia coli	Enterovirus
Shigella spp	Adenovirus
Ureaplasma spp	Influenza A
Streptococcus pyogenes	Influenza B
Salmonella spp	Parainfluenza-1
Pseudmonas aeruginosa	Parainfluenza-2
Staphylococcus aureus	Parainfluenza-3
Streptococcus pneumoniae	Respiratory syncytial virus
Streptococcus agalactiae	Parechovirus
Klebsiella spp	Enterovirus
Haemophilus influenzae	Human metapneumovirus
Neisseria meningitidis	Rubulavirus
Mycoplasma pneumoniae	Rhinovirus
Chlamydia trachomatis	Cytomegalovirus
Chlamydophila pneumoniae	Mumps virus
Bordetella pertussis	Chikungunya virus
Stenotrophomonas spp	Enterobacter
Acinetobacter spp	Alphavirus
Mycobacterium tuberculosis	Human herpesvirus
Bacillus cereus	
Elizabethkingia spp	
