# Supplementary material for: Unbiased Metagenomic Sequencing for Pediatric Meningitis in Bangladesh Reveals Neuroinvasive Chikungunya Virus Outbreak and Other Unrealized Pathogens
Source: mBio. 2019 Dec 17;10(6):e02877-19. doi: 10.1128/mBio.02877-19 (PMC6918088; doi:10.1128/mBio.02877-19)
Supplement: TABLE S5 [file mBio.02877-19-st005.pdf]

Table S5. Case-based metagenomic data derived from all sequenced samples (n=115).

Sample ID	mNGS output	Total reads	Nonhost reads	Non-host reads %	No of reads/million of the called pathogen	ERCC reads	RNA input
CHRF0052	*E. coli*	70250230	212176	0.3	465.9	41572416	17.2
CHRF0064	*S. pneumoniae*	71227742	308236	0.4	3008.2	6063522	268.7
CHRF0076	*S. pneumoniae*	63375670	98018	0.2	356.2	25658770	36.7
CHRF0088	*E. anophelis*	85955422	162734	0.2	116.5	53048838	15.5
CHRF0005	*E. hormaechei*	49785764	100862	0.2	465.9	23464986	28.0
CHRF0017	0	44873536	71250	0.2	-	25545366	18.9
CHRF0029	*E. anophelis*	54869610	373468	0.7	5758.7	46651102	4.4
CHRF0041	*K. pneumoniae*	125053116	104932	0.1	21.0	101372810	5.8
CHRF0001	0	102302082	216946	0.2	-	33395452	51.6
CHRF0013	0	12772456	93332	0.7	-	11518604	2.7
CHRF0025	*S. pneumoniae*	87017766	110194	0.1	36.0	15448776	115.8
CHRF0037	*S. pneumoniae*	95449142	9212	0.0	7.5	516	n/r
CHRF0049	*S. pneumoniae*	63460278	72664	0.1	18.8	37940022	16.8
CHRF0061	*S. pneumoniae*	102545870	74388	0.1	74.2	25325942	76.2
CHRF0073	*S. pneumoniae*	109138694	105100	0.1	554.8	6998288	364.9
CHRF0085	0	80961046	158908	0.2	-	49273376	16.1
CHRF0002	*S. pneumoniae*	141979356	2837444	2.0	19144.3	14875054	213.6
CHRF0014	*S. pneumoniae*	5138438	1520	0.0	29.7	47076	n/r
CHRF0026	0	150000000	216484	0.1	659.0	2209684	1670.2
CHRF0038	*S. pneumoniae*	131599198	57492	0.0	15.4	3102744	1035.3
CHRF0050	0	71994986	231934	0.3	-	55364152	7.5
CHRF0062	*S. pneumoniae*	150000000	47112	0.0	40.9	4619612	786.7
CHRF0074	*S. pneumoniae*	117315916	38716	0.0	5.1	1435378	2018.3
CHRF0086	*S. pneumoniae*	66783636	69142	0.1	82.0	15896928	80.0
CHRF0003	*S. pneumoniae*	56010100	88082	0.2	17.4	17328160	55.8
CHRF0015	0	45328346	208836	0.5	-	42675752	1.6
CHRF0027	*H. influenzae*	93564596	93914	0.1	56.3	43933980	28.2

Sample ID	mNGS output	Total reads	Nonhost reads	Non-host reads %	No of reads/million of the called pathogen	ERCC reads	RNA input
CHRF0039	0	88964960	47550	0.1	-	12434208	153.9
CHRF0051	*S. pneumoniae*	150000000	42228	0.0	64.5	3295142	1113.2
CHRF0063	*N. meningitidis*	83295482	338442	0.4	3.8, 3424.4	5436750	358.0
CHRF0075	*S. pneumoniae*	111766028	12132	0.0	9.3	774910	3580.8
CHRF0087	*S. pneumoniae*	69829522	30968	0.0	3.1	7217626	216.9
CHRF0004	0	60813922	237838	0.4	-	45038786	8.8
CHRF0016	0	56699154	127496	0.2	-	52922396	1.8
CHRF0028	0	39764866	166378	0.4	-	37251668	1.7
CHRF0040	0	85263302	282996	0.3	-	74509638	3.6
CHRF0053	0	46124002	83482	0.2	-	44273832	1.0
CHRF0065	0	44707958	182912	0.4	-	41350484	2.0
CHRF0077	0	75937524	102300	0.1	-	66630914	3.5
CHRF0089	0	6302354	184446	2.9	-	56770	n/r
CHRF0006	0	80267448	145860	0.2	-	77153088	1.0
CHRF0018	0	47931332	84642	0.2	-	44741152	1.8
CHRF0030	0	128845460	113374	0.1	-	120459392	1.7
CHRF0042	0	56269302	88386	0.2	-	53806038	1.1
CHRF0054	0	109272300	127002	0.1	-	103557262	1.4
CHRF0066	0	57896582	95220	0.2	-	54678742	1.5
CHRF0078	0	42345140	63466	0.1	-	39235528	2.0
CHRF0090	0	67481750	106452	0.2	-	62447376	2.0
CHRF0007	0	52481998	126918	0.2	-	49759666	1.4
CHRF0019	0	9003804	46672	0.5	-	8476152	1.6
CHRF0031	0	65032886	190336	0.3	-	62205370	1.1
CHRF0043	0	58224438	311660	0.5	-	53771664	2.1
CHRF0055	0	65419488	115050	0.2	-	61576086	1.6
CHRF0067	0	66072270	158258	0.2	-	58759328	3.1
CHRF0079	0	60221934	86664	0.1	-	52120452	3.9
CHRF0091	0	68421536	123050	0.2	-	62355960	2.4

Sample ID	mNGS output	Total reads	Nonhost reads	Non-host reads %	No of reads/million of the called pathogen	ERCC reads	RNA input
CHRF0008	0	56664264	127858	0.2	-	47826772	4.6
CHRF0020	0	75031264	545870	0.7	-	64841106	3.9
CHRF0032	0	52942812	94000	0.2	-	50417636	1.3
CHRF0044	0	74679170	126652	0.2	-	70394724	1.5
CHRF0056	0	58958602	100272	0.2	-	52111098	3.3
CHRF0068	0	58263836	81770	0.1	-	53534916	2.2
CHRF0080	0	58480150	77640	0.1	-	48353780	5.2
CHRF0092	0	51999596	83734	0.2	-	46960542	2.7
CHRF0009	0	42981288	122776	0.3	-	39365830	2.3
CHRF0021	0	38396862	145968	0.4	-	35594906	2.0
CHRF0033	0	50925620	68302	0.1	-	45749064	2.8
CHRF0045	0	50455298	80706	0.2	-	48657850	0.9
CHRF0057	0	50024148	95218	0.2	-	46905102	1.7
CHRF0069	0	45846480	59750	0.1	-	42479872	2.0
CHRF0096	0	49342992	69600	0.1	-	46646414	1.4
CHRF0000	0	135087088	156310	0.1	-	130150782	0.9
CHRF0081	0	64453194	38766	0.1	-	31709728	25.8
CHRF0093	0	49670048	99216	0.2	-	43264818	3.7
CHRF0010	*Enterovirus B*	61327650	70042	0.1	9.3	22230004	44.0
CHRF0022	0	118348946	181994	0.2	-	70802894	16.8
CHRF0034	0	60169132	112136	0.2	-	13310622	88.0
CHRF0046	0	42195836	158576	0.4	-	25483100	16.4
CHRF0058	*M. tuberculosis*	65072802	34314	0.1	9.5	3104784	499.0
CHRF0070	*B. cereus*	69060574	98978	0.1	247.4	5797930	272.8
CHRF0082	*S. enterica*	60637346	98414	0.2	97.8	19935498	51.0
CHRF0094	CHIKV	61336096	412890	0.7	5495.9	28094424	29.6
CHRF0011	Mumps	60553510	25850	0.0	0.7	7677666	172.2
CHRF0023	0	53928028	285306	0.5	-	38094584	10.4

Sample ID	mNGS output	Total reads	Nonhost reads	Non-host reads %	No of reads/million of the called pathogen	ERCC reads	RNA input
CHRF0035	Human herpes virus 6 (manual)	96343858	16208	0.0	-	1643200	1440.8
CHRF0047	0	57593626	54800	0.1	-	6349378	201.8
CHRF0059	*S. maltophilia*	85986872	1362544	1.6	3240.9	38318362	31.1
CHRF0071	CHIKV	58234020	85112	0.1	242.8	23316766	37.4
CHRF0083	0	139233556	48796	0.0	-	286176	n/r
CHRF0095	0	61324940	84592	0.1	-	19572240	53.3
CHRF0012	CHIKV	8760674	56784	0.6	1830.1	3462238	38.3
CHRF0024	0	98585738	86354	0.1		20945142	92.7
CHRF0036	Mumps (manual)	72872708	62864	0.1	0 rpm, 2 total reads	8675260	185.0
CHRF0048	0	77389688	62590	0.1	-	58257176	8.2
CHRF0060	0	81192516	193074	0.2	-	70471964	3.8
CHRF0072	0	150000000	89218	0.1	-	31927140	92.1
CHRF0084	0	59849024	57728	0.1	-	49233396	5.4
CHRF0097	CHIKV	150000000	120342	0.1	24.9	130431532	3.8
CHRF0098	CHIKV	150000000	187842	0.1	20.9	141728870	1.5
CHRF0099	CHIKV	150000000	414592	0.3	6423.7	136277954	2.5
CHRF0100	CHIKV	150000000	311012	0.2	8.0	95075520	14.4
CHRF0101	CHIKV	150000000	1632694	1.1	20200.8	96521140	13.9
CHRF0102	CHIKV	150000000	436436	0.3	111.5	113727008	8.0
CHRF0103	CHIKV	150000000	381190	0.3	3254.2	121280236	5.9
CHRF0104	CHIKV	150000000	371278	0.2	106.0	116330080	7.2
CHRF0105	CHIKV	150000000	228930	0.2	881.3	105689416	10.5
CHRF0106	CHIKV	150000000	359446	0.2	4169.4	117445236	6.9
CHRF0107	CHIKV	150000000	171530	0.1	966.1	138854562	2.0
CHRF0108	CHIKV	150000000	241328	0.2	248.9	131571546	3. 5
CHRF0109	CHIKV	150000000	157336	0.1	565.6	138399578	2.09
CHRF0110	CHIKV	150000000	6795359	4.5	37386.1	16650300	200.3
CHRF0111	CHIKV	150000000	223136	0.1	1065.6	136500544	2.5

Sample ID	mNGS output	Total reads	Nonhost reads	Non-host reads %	No of reads/million of the called pathogen	ERCC reads	RNA input
CHRF0112	CHIKV	150000000	25772	0.0	3.0	319182	n/r
CHRF0113	CHIKV	150000000	1357528	0.9	34.8	72993550	26.7
CHRF0114	0	150000000	231476	0.2	-	139254112	1.9

n/r: the RNA input could not be back-calculated from ERCC counts.
