# Supplementary material for: Unbiased Metagenomic Sequencing for Pediatric Meningitis in Bangladesh Reveals Neuroinvasive Chikungunya Virus Outbreak and Other Unrealized Pathogens
Source: mBio. 2019 Dec 17;10(6):e02877-19. doi: 10.1128/mBio.02877-19 (PMC6918088; doi:10.1128/mBio.02877-19)
Supplement: TEXT S2 [file mBio.02877-19-s0002.pdf]

Method S2. Phylogenetic analysis of Chikungunya virus strains responsible for the meningitis outbreak in Bangladesh, 2017.

To study the phylogeny of the Chikungunya virus (CHIKV), BLASTn was used to extract all complete CHIKV genomes that had greater then 85% identity to the draft genomes assembled using SPAdes v3.11.1.(10) These genomes were then aligned using the default settings in MUSCLEv3.8.1551.(11) Annotation from one of the NCBI genomes (accession number: HM045823) was used to divide the genome into coding, and non-coding regions, and ModelTest-NGv0.1.5 was used to identify the best-fitting evolutionary models for each genomic region. Using the best-fitting models for evolution for each genomic region, we reconstructed a maximum-likelihood phylogeny using RAxML-ng v0.6.0 using default settings.(12) The maximum likelihood phylogeny was used to select a lineage of most closely related NCBI sequences to the assembled chikungunya virus genomes. We then created a time-resolved phylogeny for this lineage in BEAST v1.10.1 using substitution models selected using ModelTest-NG a strict molecular clock for all genomic sites, and selected the Bayesian skyline model as the tree prior.(13, 14). Two separate BEAST runs of 300 million Markov chain Monte Carlo iterations sampling every 50,000 iterations were used to explore the posterior distribution of phylogenies and evolutionary parameters. Convergence was visually checked after removing the first 50% of samples, and samples from the two chains were merged. A maximum clade credibility phylogeny was created using median node heights. Using the above described method, another time-resolved phylogeny of the Bangladesh CHIKV was generated using the genomes assembled earlier and E1 viral structural glycoprotein sequence from 2016-2017 South Asian cluster [Pakistan 2016 CHIKV outbreak (accession number: MF774613—MF774619, MF74074—MF74081), Bangladesh 2017 outbreak (accession number: MG697262—MG697282), Australia (accession number: KY751908), Italy (accession number: MG049915) Hong-Kong (accession number: MF503628) and India (accession number: KY057363)].

Accession number of Enterovirus B, CHRF0010, is MK468615 of the CHIKV genomes are CHRF_0071: MK468608; CHRF_0103: MK468609; CHRF_0094: MK468610; CHRF_0099: MK468611; CHRF_0101: MK468612; CHRF_0106: MK468613; CHRF_0108: MK468614; CHRF_0110: MK468615; CHRF_0104: MK468616; CHRF_0105: MK468617; CHRF_0107: MK468618; CHRF_0109: MK468619; CHRF_0012: MK468620; CHRF_0111: MK468621; CHRF_0112: MK468622; CHRF_0098: MK468623; CHRF_0100: MK468624; CHRF_0097: MK468625; CHRF_0102: MK468626; CHRF_0113: MK468627; CHRF_0012: MK468628; CHRF_0012: MK468629

References:

10. Bankevich A, Nurk S, Antipov D, Gurevich AA, Dvorkin M, Kulikov AS, Lesin VM, Nikolenko SI, Pham S, Prjibelski AD, Pyshkin A V., Sirotkin A V., Vyahhi N, Tesler G, Alekseyev MA, Pevzner PA. 2012. SPAdes: A New Genome Assembly Algorithm and Its Applications to Single-Cell Sequencing. J Comput Biol 19.5:455-477.
11. Edgar RC. 2004. MUSCLE: multiple sequence alignment with high accuracy and high throughput. Nucleic Acids Res 32:1792–7.
12. Kozlov A, Darriba D, Flouri T, Morel B, Stamatakis A. 2018. RAxML-NG: A fast, scalable, and user-friendly tool for maximum likelihood phylogenetic inference. bioRxiv 447110.
13. Drummond AJ, Rambaut A. 2007. Bayesian evolutionary analysis by sampling trees. BMC Evol Biol 7:214.
14. Darriba D, Posada D, Kozlov AM, Stamatakis A, Morel B, Flouri T. 2019. ModelTest-NG: a new and scalable tool for the selection of DNA and protein evolutionary models. BioRxiv 612903.
